# BopN is a Gatekeeper of the *Bordetella* Type III Secretion System

**DOI:** 10.1128/spectrum.04112-22

**Published:** 2023-04-10

**Authors:** Kevin Munoz Navarrete, Ladislav Bumba, Tatyana Prudnikova, Ivana Malcova, Tania Romero Allsop, Peter Sebo, Jana Kamanova

**Affiliations:** a Laboratory of Infection Biology, Institute of Microbiology of the Czech Academy of Sciences, Prague, Czech Republic; b Laboratory of Molecular Biology of Bacterial Pathogens, Institute of Microbiology of the Czech Academy of Sciences, Prague, Czech Republic; c Faculty of Science, University of South Bohemia in Ceske Budejovice, Ceske Budejovice, Czech Republic; Centre national de la recherche scientifique, Aix-Marseille Université

**Keywords:** BopN, *Bordetella*, gatekeeper, type III secretion system

## Abstract

The classical *Bordetella* species infect the respiratory tract of mammals. While B. bronchiseptica causes rather chronic respiratory infections in a variety of mammals, the human-adapted species B. pertussis and *B. parapertussis*_HU_ cause an acute respiratory disease known as whooping cough or pertussis. The virulence factors include a type III secretion system (T3SS) that translocates effectors BteA and BopN into host cells. However, the regulatory mechanisms underlying the secretion and translocation activity of T3SS in bordetellae are largely unknown. We have solved the crystal structure of BopN of B. pertussis and show that it is similar to the structures of gatekeepers that control access to the T3SS channel from the bacterial cytoplasm. We further found that BopN accumulates at the cell periphery at physiological concentrations of calcium ions (2 mM) that inhibit the secretion of BteA and BopN. Deletion of the *bopN* gene in B. bronchiseptica increased secretion of the BteA effector into calcium-rich medium but had no effect on secretion of the T3SS translocon components BopD and BopB. Moreover, the Δ*bopN* mutant secreted approximately 10-fold higher amounts of BteA into the medium of infected cells than the wild-type bacteria, but it translocated lower amounts of BteA into the host cell cytoplasm. These data demonstrate that BopN is a *Bordetella* T3SS gatekeeper required for regulated and targeted translocation of the BteA effector through the T3SS injectisome into host cells.

**IMPORTANCE** The T3SS is utilized by many Gram-negative bacteria to deliver effector proteins from bacterial cytosol directly into infected host cell cytoplasm in a regulated and targeted manner. Pathogenic bordetellae use the T3SS to inject the BteA and BopN proteins into infected cells and upregulate the production of the anti-inflammatory cytokine interleukin-10 (IL-10) to evade host immunity. Previous studies proposed that BopN acted as an effector in host cells. In this study, we report that BopN is a T3SS gatekeeper that regulates the secretion and translocation activity of *Bordetella* T3SS.

## INTRODUCTION

The closely related classical *Bordetella* species, B. pertussis, *B. parapertussis*, and B. bronchiseptica, cause respiratory infections in mammals. The strictly human-adapted pathogen B. pertussis is the main causative agent of whooping cough or pertussis, a highly transmissible and acute respiratory infectious illness that has recently resurged in vaccinated populations (1). *B. parapertussis*_HU_ causes a milder form of whooping cough in humans, and its other lineages infect ovines. In contrast, B. bronchiseptica has a broad host range and infects a variety of mammalian species, eliciting pathologies ranging from typical chronic respiratory infections to acute illnesses, such as the kennel cough in dogs, atrophic rhinitis in swine, snuffles in rabbits, and bronchopneumonia in guinea pigs (2, 3). The 3 classical *Bordetella* species produce numerous virulence factors involved in colonization of host airways, immune system evasion, and transmission to new hosts. These include adhesins and toxins, such as the highly conserved adenylate cyclase and dermonecrotic toxins (4). A type III secretion system (T3SS) was shown to specifically contribute to the persistence of B. bronchiseptica in the host respiratory tract (5–7), whereas the role of the T3SS in infections by B. pertussis remains to be clarified (8).

The T3SS of bordetellae is a multicomponent protein-export apparatus that injects effector proteins BteA and BopN directly from the *Bordetella* cytosol into host cells (9, 10). The process requires the assembly of a pore in the host plasma membrane, formed by the two *Bordetella* translocator proteins BopB and BopD (11, 12). Further, it depends on one of the most abundantly secreted T3SS substrates, the Bsp22 protein that is thought to act as a unique tip of *Bordetella* injectisome connecting the T3SS needle and the translocon pore (13). Upon injection into host cells, the 69-kDa BteA protein, also known as BopC, localizes to the lipid microdomains (rafts) at the inner face of the cell plasma membrane and triggers a rapid death of eukaryotic cells via an unknown mechanism (9, 14–17). Interestingly, the BteA protein from B. pertussis exhibits a much lower cytotoxic activity than BteA of B. bronchiseptica, due to the insertion of an additional alanine residue at position 503 (A503), which most likely represents an evolutionary adaptation of B. pertussis to the acute infection lifestyle in the human host (18). The other T3SS-injected protein, BopN, has been reported to interfere with *B. bronchiseptica*-induced NF-κB signaling, provoking upregulation of the anti-inflammatory cytokine IL-10 and thereby undermining host protective immune responses (10, 19). In addition, BopN has been described to promote BteA-mediated cytotoxicity of B. bronchiseptica on rat L2 pulmonary epithelial cells but not on mouse DC2.4 dendritic cells (10, 19). No mechanistic details of the BopN action(s) are known. However, the amino acid sequence of the 365 residues of the BopN protein shows 19% identity with the *Yersinia* protein YopN, which regulates the biogenesis of T3SS and belongs to the family of SctW proteins known as gatekeepers (6, 20, 21).

T3SS biogenesis in Gram-negative bacteria is a tightly regulated process in which substrates are secreted in a specific order. After Sec-dependent assembly of the basal body, type III-mediated secretion begins with the release of the early substrates that form the inner rod and needle, followed by the transport of intermediate substrates that include the needle tip and translocator proteins, and finally, secretion of the late substrates, the effector proteins (22, 23). The effectors are translocated only when the needle tip senses the host cell. However, contact with the host cell can be artificially mimicked by various chemical signals, such as low concentration of calcium ions in *Yersinia* and enteropathogenic Escherichia coli (24–26), pH shifts in Salmonella pathogenicity island 2 (SPI-2) T3SS (27), Congo red in *Shigella* (28), and high potassium ion concentration in *Vibrio* (29), all triggering effector secretion. Proteins that prevent the premature release of effectors under secretion-restrictive conditions (before contact with the host cell or without an artificial trigger) serve as gatekeepers and are referred to as SctW in the unified nomenclature of Sct proteins. The members of this family include SepL in enteropathogenic *Esterichia coli* (EPEC), MxiC in *Shigella*, InvE and SsaL in Salmonella, CopN in Chlamydia, and previously mentioned *Yersinia* protein YopN (22, 23). These proteins interact with the SctV type of proteins of the T3SS export apparatus, and it has been suggested that they block secretion of effectors either by forming a physical barrier, or by having allosteric effects on SctV function, while supporting the loading of translocator-chaperone complexes (30–33). Following an activation signal (contact with the host cell and/or an artificial trigger), the gatekeeper is released from SctV and effector secretion is initiated (31, 33). Inactivation of the gatekeeper causes deregulation of effector secretion independently of activation conditions and accounts for defects in targeted effector delivery. For example, *Yersinia* mutants lacking the gatekeeper YopN secrete effector proteins even in the presence of 2 mM Ca^2+^ (secretion-restrictive conditions) and display leakage of effectors into cell medium during cell infection (34–37). Interestingly, the fate of the gatekeeper after its release from SctV varies among bacterial species. It is either degraded, as in the case of the SsaL gatekeeper of Salmonella SPI-2 (27), or it is translocated into host cells, like YopN of *Yersinia*, MxiC of Shigella flexneri, or CopN of Chlamydia (36, 38, 39). Gatekeepers are not thought to have effector functions when injected into the host cell, except Chlamydia CopN that has been shown to cause cell cycle arrest in G2/M phase by inhibiting tubulin polymerization (40–42).

In this study, we addressed the mechanism underlying the secretion and translocation activity of T3SS in bordetellae. Specifically, we determined the structure of the BopN protein and investigated its role as a gatekeeper of the T3SS in B. bronchiseptica.

## RESULTS

### BopN adopts a conserved structure of the type III secretion gatekeepers.

**FIG 1:**
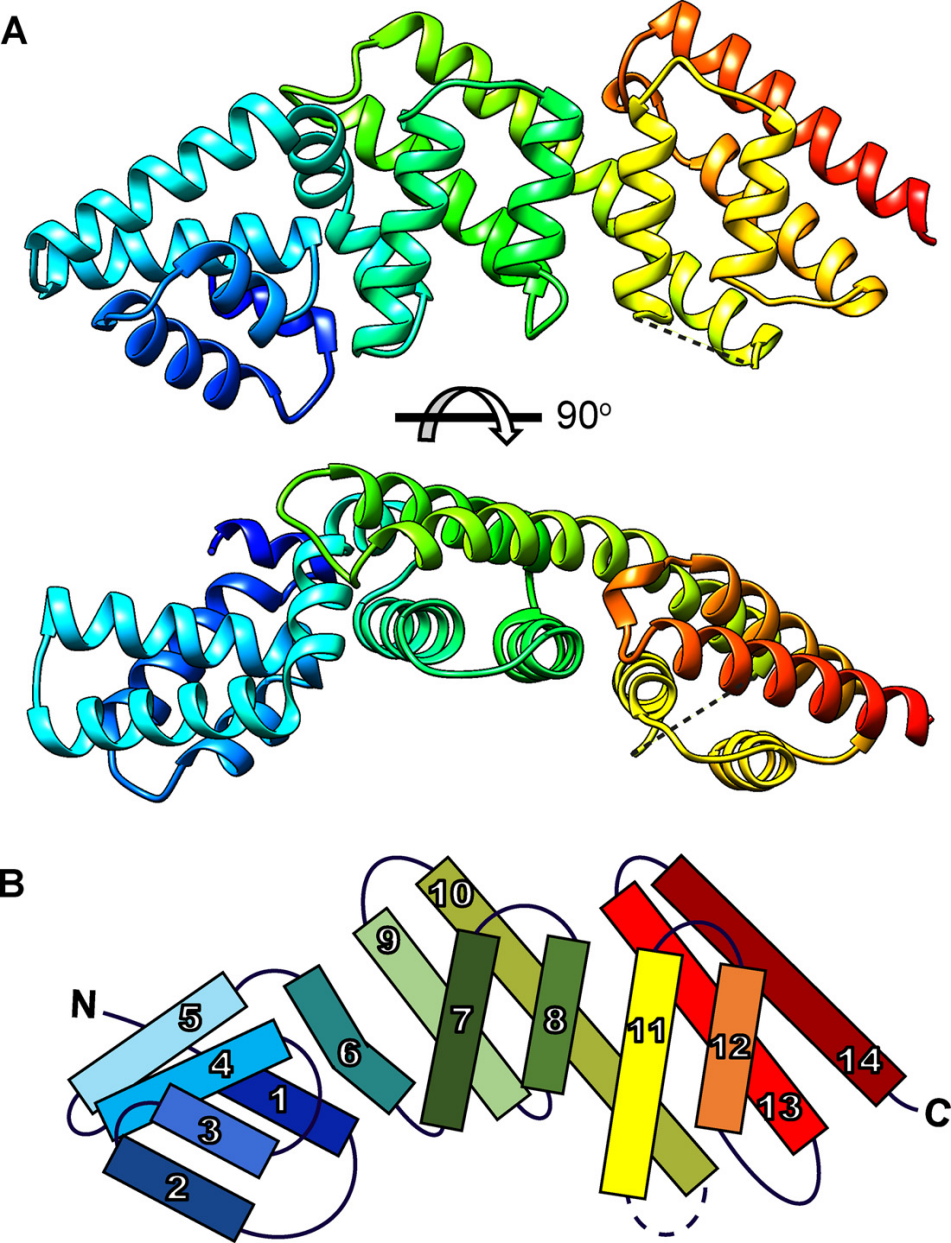
Structure of the BopN protein. (A) Ribbon representation of BopN residues 83 to 365. The bottom structure is a 90° rotation around the horizontal axis of the BopN structure shown above. The broken line between BopN residues 276 and 282 denotes the disordered region of the structure. This figure was generated by Chimera 1.14rc. (B) Topology diagram of the BopN protein. The helices 1 to 14 are arranged in 3 X-bundle domains. BopN domains 1 and 2 are connected by a kinked helix 6, whereas domains 2 and 3 are connected by the rigid central helix 10 and a region with no density in the electron map.

To gain insight into BopN function in the *Bordetella* injectisome, we first determined its structure by X-ray crystallography and identified structures with a similar fold. To obtain a stable protein fragment of BopN suitable for crystallization experiments, we performed limited proteolysis of the full-length BopN protein (BopN 1 to 365) of B. pertussis Tohama I by trypsin. A stable protein fragment with an intact mass of 31,869 Da was obtained corresponding to a truncated BopN protein lacking the first 68 residues (BopN 69 to 365). The protein crystallized in a tetragonal (*P*4_2_22) space group with a single molecule per asymmetric unit and its structure was determined with a 1.95 Å resolution (Table 1). As shown by a ribbon representation (Fig. 1A) and by a topology diagram (Fig. 1B), BopN is an elongated protein consisting of three X-bundle domains. The N-terminal domain comprises 5 helices 1 to 5 and includes residues 83 to 161. Residues 69 to 82 are not visible, probably due to conformational flexibility or partial degradation. A kinked helix 6 (residues 162 to 180) connects the N-terminal domain to a central domain consisting of parallel helices 7 and 8 located on top of helices 6, 9, and 10. The C-terminal domain consists of parallel helices 11 and 12, overlying helices 10, 13, and 14. It is connected to the central domain by a shared rigid central helix 10 (residues 243 to 274) and a region (residues 275 to 281) that lacks density in the electron map, indicating loop flexibility.

**TABLE 1:** X-ray diffraction data and collection statistics^*a*^

Data processing statistics	
Diffraction source	MX 14.1, BESSY II
Wavelength (Å)	0.9184
Temp (K)	100
Detector	PILATUS3 S 2M
Crystal-detector distance (mm)	297.07
Rotation range per image (°)	0.1
Total rotation range (°)	180
Exposure time per image (s)	0.6
Space group	*P*4_2_22 (93)
Cell dimensions	
a, b, c (Å)	84.86, 84.86, 102.57
Α, β, γ (°)	90.0, 90.0, 90.0
Resolution (Å)	43.93-1.95 (2.00-1.95)
Redundancy	8.88
No. of reflections	
Total	236,216 (36,301)
Unique	26,601 (4,088)
Completness (%)	99.91 (99.85)
R_meas_ (%)	10.4 (12.7)
I/σ	18.7 (1.75)
CC half (%)	99 (73)
Overall B factor from Wilson plot (Å^2^)	36.5
Refinement statistics	
Resolution range (Å)	43.93–1.95
No. of reflections, working set	26586
R value^*b*^ (%)/R_free_^*c*^ (%)	17.97/20.71
No. of atoms:	
Protein	2206
Water	197
Glycerol	1
PEG	1
No. of ions:	
Sodium/Calcium ions	1/1
Acetate ion	1
Root mean square deviation	
Bond lengths (Å)	0.017
Bond angles (°)	1.878
Avg B factors (Å^2^) Overall	46.01
Ramachandran plot	
Most favored region (%)	98.7
Allowed region (%)	100.0
PDB code	7YYG

a The data in parentheses refer to the highest-resolution shell.

b R value = ‖F_o_| − |F_c_‖/|F_o_|, where F_o_ and F_c_ are the observed and calculated structure factors, respectively.

c R_free_ is equivalent to R value but is calculated for 5% of the reflections chosen at random and omitted from the refinement process.

Structural homology search using the algorithm DALI (43) revealed that the characterized T3SS gatekeepers, namely, *Shigella* MxiC (44), Chlamydia CopN (42), EPEC SepL (45), and the *Yersinia* YopN-TyeA complex (46), scored highest (Table 2). Similar to BopN, these proteins exhibit a well-conserved architecture with three four-helix X-bundle domains. However, in the case of YopN, the three-X-bundle domain structure is achieved only after the binding of the TyeA protein to a C-terminal region of YopN (46). The relative orientation of the X-bundle domains in different molecules slightly varies, yielding different overall molecular shapes. When the BopN molecule is superimposed on the structurally characterized gatekeepers (see Fig. S1), the overall shapes and orientations of the X-bundle domains also differ, with the most noticeable difference being the realignment of the N-terminal domain of BopN. This is also reflected in the DALI algorithm Z-scores for BopN (Table 2), which are in good agreement with previously reported Z-scores within the SctW family (45). These are indicative of significant similarity but are typically not high enough to define a strong match between the structures (Z-score > 24 would define a strong match for BopN, (47)).

**TABLE 2:** Proteins structurally related to BopN^*a*^

Protein	PDB/chain	z-score	RMSD (Å)	No. of Cα atoms aligned	No. of residues	% id of aligned residues
*Shigella* MxiC	2vj5-B	20.9	4.5	265	283	17
Chlamydia CopN	6gx7-H	20.2	7.3	264	290	17
E. coli SepL	5c9e-B	14.1	4.0	182	263	15
*Yersinia* TyeA	1xl3-D	12.8	1.7	83	85	23
*Yersinia* YopN	1xl3-B	12.7	3.0	175	206	19

a Structure of the BopN protein was correlated using DALI (distance matrix alignment) algorithm (43). The highest-ranking structures are shown.

In summary, BopN structure resembles the structures of other gatekeepers, suggesting that BopN may regulate translocation activity in *Bordetella*, similar to the SctW members of the T3SS injectisomes of other bacteria.

### Inactivation of BopN partially deregulates type III secretion in calcium-rich medium.

It was next important to determine whether BopN serves as the T3SS gatekeeper in *Bordetella* and prevents premature secretion of BteA under secretion-restrictive conditions. Since BopN protein sequences of B. pertussis Tohama I and B. bronchiseptica RB50 exhibit only a single amino acid difference, namely, a P112T substitution in the loop connecting helices 3 and 4, we chose to perform the subsequent experiments with B. bronchiseptica, for which the importance of T3SS function in host respiratory tract infections has been demonstrated (5–7). Moreover, B. bronchiseptica has the T3SS constitutively active in the low calcium concentration (0.1 mM Ca^2+^) SS medium and its secreted BteA effector is readily detectable in culture supernatants (9). We further hypothesized that as for other T3SS, a low concentration of calcium ions might trigger effector secretion by B. bronchiseptica (24–26), and a millimolar concentration of calcium ions in the growth medium would create secretion-restrictive conditions and prevent BteA secretion.

**FIG 2:**
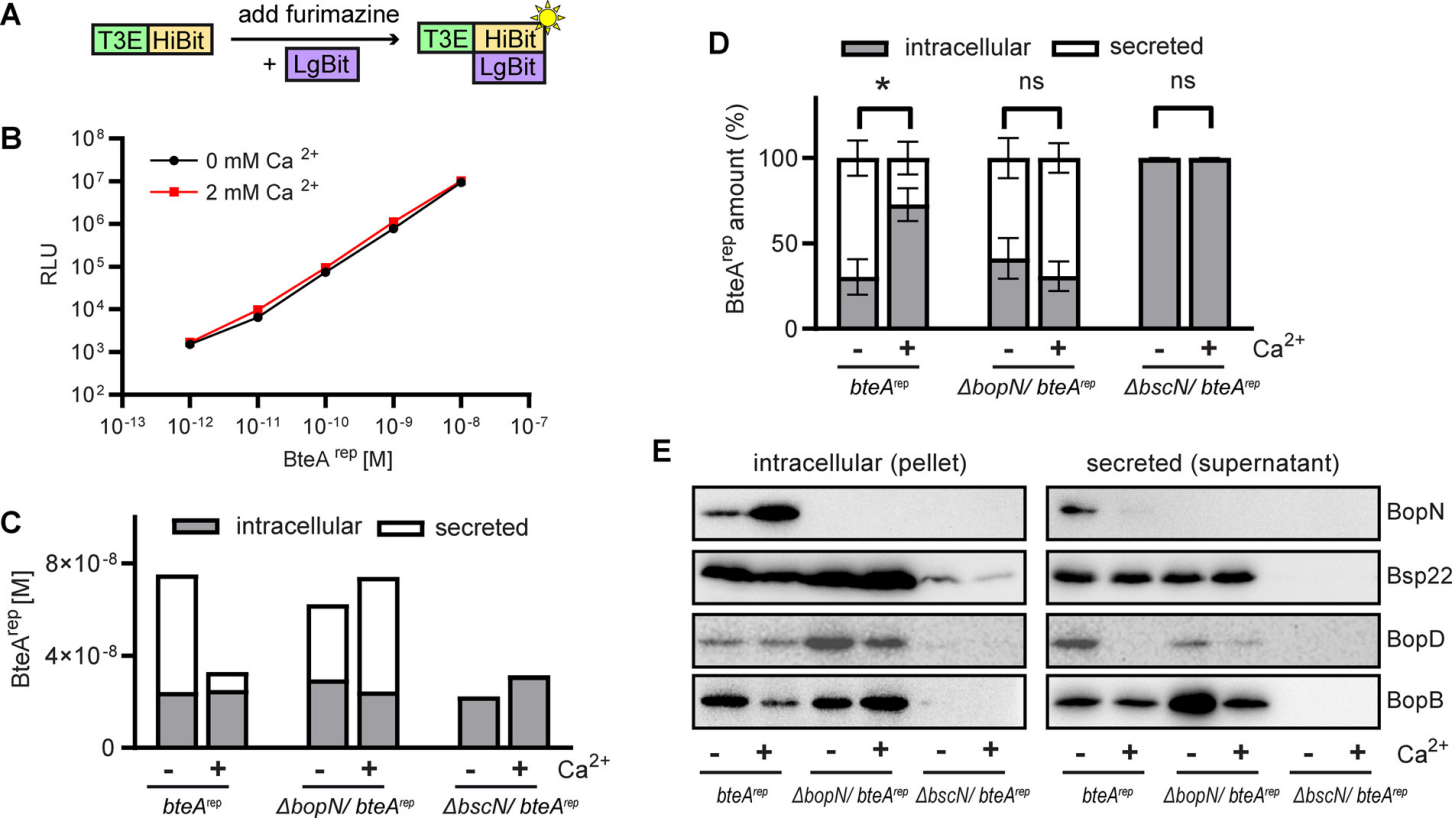
Inactivation of BopN leads to a partial deregulation of the type III secretion in calcium-rich medium. (A) Schematic representation of HiBiT-LgBit functional complementation for quantification of protein amounts. The HiBit peptide fused to the protein of interest, e.g., type III effector, T3E, binds to added LgBit. The complex generates a luminescent signal in the presence of furimazine substrate. (B) Linearity of luminescence generated by BteA^rep^-LgBit complementation. Luminescence was measured after the addition of LgBit and furimazine substrate to the indicated amounts of purified recombinant BteA^rep^ in the absence or presence of 2 mM Ca^2+^. Shown are mean values ± SD of triplicate wells from a representative experiment of 3 experiments performed. (C) and (D) BopN controls secretion of BteA. The amounts of intracellular and secreted BteA^rep^ in exponential cultures grown in the absence or presence of 2 mM Ca^2+^ were determined by luminescence measurements after appropriate dilution. In (C), the absolute amounts of BteA^rep^ in fractions of the representative culture are shown. In (D), the amount of BteA^rep^ in the fraction was expressed as % of total BteA^rep^ in the culture. Values represent the means ± SD of 3 independent experiments. Statistical analysis was performed using an unpaired two-tailed *t* test; * *P < *0.05; ns, not significant. (E) BopN does not regulate secretion of the tip protein Bsp22 and translocators BopD and BopB. Supernatants and pellets from overnight cultures of the b*teA*^rep^ strain and its derivatives were analyzed by immunoblotting with anti-BopN (BopN, 1:10,0000), anti-Bsp22 (Bsp22, 1:10,000), anti-BopD (BopD, 1:10,0000), and anti-BopB (BopB, 1:10,000) antisera. Results are representative of 3 independent experiments.

To quantitatively monitor the BteA content in bacterial cells and the secreted fractions, we implemented a split-luciferase system (48–50) and used a nontoxic *bteA*^rep^ reporter strain of B. bronchiseptica RB50 in which the N-terminal 130-amino acid segment of BteA was tagged at the C-terminus with a HiBiT peptide. As shown schematically in Fig. 2A, the split-luciferase reporter system exploits a high-affinity functional complementation between a small 11-amino acid tag called HiBiT and a larger 18-kDa fragment LgBit. The complex exerts a luciferase activity and emits light in the presence of added furimazine substrate. Therefore, the amounts of BteA^rep^ in the intracellular and secreted fractions can be easily quantified upon the addition of the LgBit fragment and furimazine. Indeed, as shown by dilution series of purified recombinant BteA^rep^ protein in the presence of LgBit fragment in excess, the generated luminescence signal is linear over several orders of magnitude of BteA^rep^ concentrations and is not affected by the concentration of calcium ions (Fig. 2B). Importantly, as shown in Fig. 2C, analysis of BteA^rep^ amounts in late exponential cultures of *bteA*^rep^ revealed that growth in calcium-rich (2 mM) medium decreased the secreted amounts of BteA^rep^ without affecting its intracellular levels, compared to medium without calcium ions. Indeed, BteA^rep^ was secreted rather inefficiently in calcium-rich medium, with a BteA_out_/BteA_in_ ratio of ~ 0.3. In contrast, in the absence of calcium ions, the ratio of BteA_out_/BteA_in_ was ~ 2.3, as further shown in Fig. 2D. Hence, in the presence of 2 mM calcium ions, BteA secretion decreased 7-fold, suggesting that calcium-rich media creates secretion-restrictive conditions. As expected, negligible amounts of BteA^rep^ were detected in the culture medium of the type III secretion-deficient Δ*bscN*/*bteA*^rep^ mutant (deleted BscN ATPase essential for T3SS function), showing that BteA^rep^ excretion into supernatants specifically depends on T3SS functionality. Importantly, upon *bopN* gene deletion from the genome of the reporter strain, BteA^rep^ secretion was not blocked in the calcium-rich medium anymore (Fig. 2C and D). Indeed, the Δ*bopN*/*bteA*^rep^ strain culture yielded a BteA_out/_BteA_in_ ratio of ~ 2.3 even in calcium-rich medium, whereas this ratio was ~ 1.3 in the absence of calcium ions. Moreover, consistent data were obtained at all phases of the B. bronchiseptica growth curve (early exponential, late exponential, and stationary growth), as shown in Fig. S2, demonstrating that BopN prevents secretion of BteA under secretion-restrictive conditions.

We next characterized the secretion of other *Bordetella* T3SS components under secretion-restrictive (2 mM Ca^2+^) and secretion-permissive (0 mM Ca^2+^) conditions, focusing on the role of the BopN protein in secretion of the T3SS tip protein Bsp22 and of the translocator proteins BopD and BopB. Interestingly, immunoblot analysis of culture supernatants and pellets from overnight (early stationary) cultures revealed that compared to secretion-permissive conditions, also BopN secretion was prevented in secretion-restrictive medium (Fig. 2E and Fig. S3). In contrast, no significant impact on secretion of the tip protein Bsp22 and of the translocator proteins BopB and BopD was observed in secretion-restrictive medium or upon inactivation of the *bopN* gene. Whereas the trends of BopN and Bsp22 detection in intracellular and secreted fractions were consistent and highly reproducible, a high variation in detected amounts of BopB and BopD proteins between fractions was observed for unclear reasons (Fig. 2E and Fig. S3).

In summary, hence, BopN exerted a control over the secretion of the BteA effector but not over the secretion of the Bsp22, BopB and BopD components of the injectisome. Inactivation of the *bopN* gene then led to enhanced secretion of BteA in secretion-restrictive culture conditions (2 mM Ca^2+^). Furthermore, data also indicated that secretion of the BopN protein itself was prevented under secretion-restrictive conditions.

### BopN responds to low calcium ion concentration and localizes to *Bordetella* cell periphery in calcium-rich medium.

**FIG 3:**
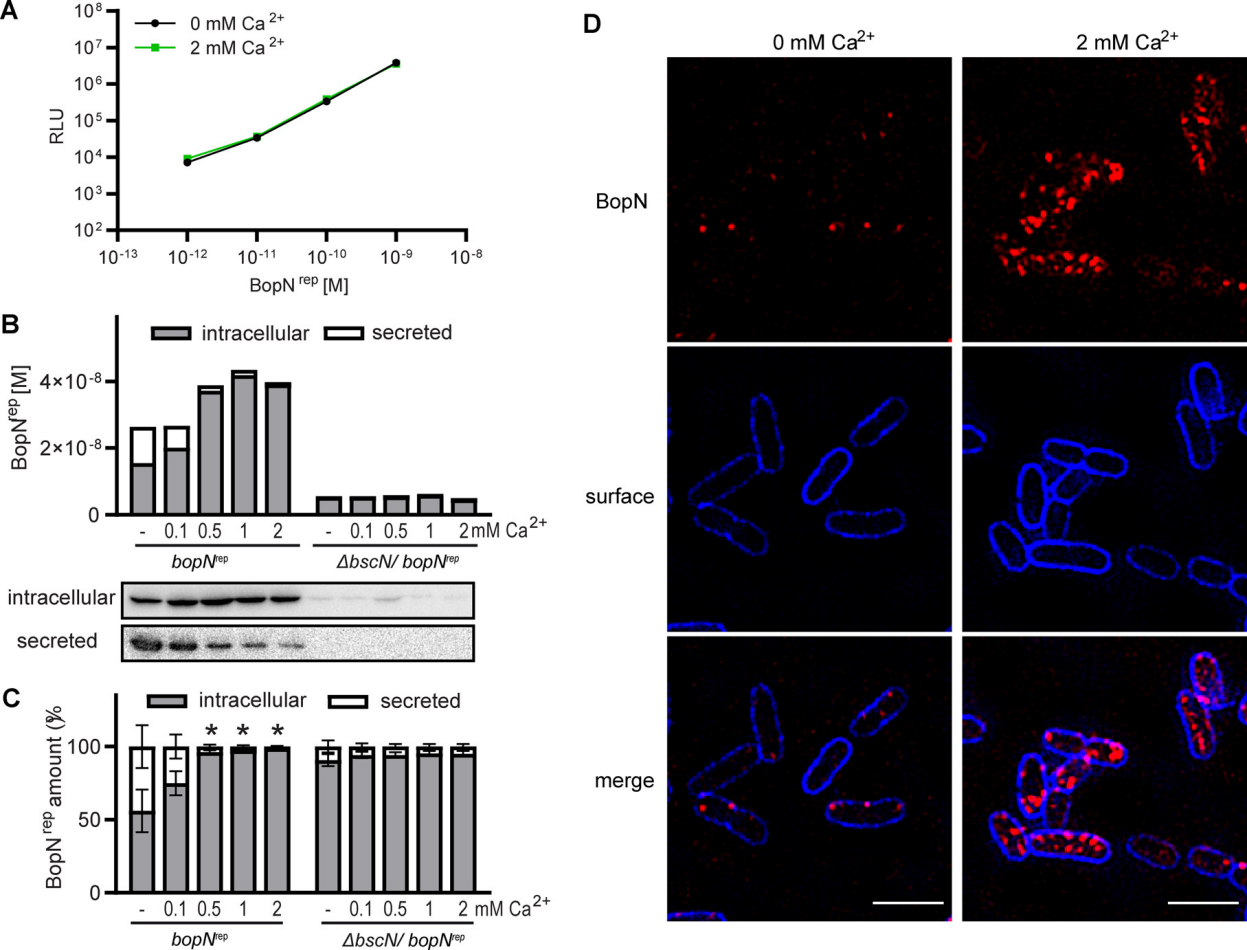
BopN responds to low Ca^2+^ concentration and localizes to the cell periphery. (A) Linearity of luminescence generated by BopN^rep^-LgBit complementation. Luminescence was measured after the addition of LgBit and furimazine substrate to the indicated amounts of purified recombinant BopN^rep^ in the presence or absence of 2 mM Ca^2+^. Shown are mean values ± SD of triplicate wells from a representative experiment of 3 experiments performed. (B) and (C) Calcium-rich medium prevents BopN secretion. Cells of the *bopN*^rep^ and the secretion-deficient Δ*bscN*/*bopN*^rep^ derivative were incubated at the indicated concentration of Ca^2+^ for 90 min. The amounts of intracellular and secreted BopN^rep^ were determined by luminescence measurements after appropriate dilution. In (B) the absolute amounts and immunoblot detection of BopN^rep^ in fractions of the representative culture are shown. In (C), the amount of Bop^rep^ in the fraction was expressed as % of total BopN^rep^ in the culture. Values represent the mean ± SD of 3 independent experiments. Significant differences (*, *P < *0.05, unpaired two-tailed *t* test) between BopN^rep^ secreted in the absence of calcium and the corresponding culture at the indicated calcium ion concentration are marked. (D) BopN^rep^ localizes to the periphery of *Bordetella* cells in calcium-rich medium. Cells of *bopN*^rep^ were fixed on polylysine-coated coverslips after incubation for 90 min in the absence or presence of 2 mM Ca^2+^. BopN^rep^ was visualized with an anti-SPOT nanobody conjugated to ATTO594, whereas the outer surface of the bacteria was stained with a rabbit anti-*Bordetella* serum and detected with the DyLight 405-labeled anti-rabbit IgG conjugate. A single focal plane of a Z-stack is shown. Fluorescence images are representative of 3 independent experiments. Scale bar represents 2 μm.

To corroborate the mechanism of BopN action, we next constructed a *bopN^rep^* reporter strain of B. bronchiseptica RB50 expressing a chromosomally-encoded BopN^rep^ protein tagged at its C-terminus with the peptide HiBiT-3xFLAG-SPOT. The resulting bopN^rep^ strain exhibited the same T3SS-dependent cytotoxicity against A549 lung epithelial cells as the wild-type strain (Fig. S4A), demonstrating that the tagging of BopN did not affect T3SS injectisome function. As in the case of recombinant BteA^rep^, dilution series of purified recombinant BopN^rep^ to which LgBit was added in excess showed that binding of BopN^rep^ to LgBit produced a luminescence signal that was linear over several orders of magnitude of the BopN concentrations and was independent of calcium ions (Fig. 3A), thus allowing quantitative monitoring of BopN^rep^ secretion in response to calcium ions. To this end, *bopN*^rep^ bacteria grown in calcium-rich medium (2 mM Ca^2+^) were transferred into medium with various Ca^2+^ concentrations (0, 0.1, 0.5, 1, and 2 mM) and incubated for 90 min. Subsequently, the amounts of secreted and intracellular BopN^rep^ protein were determined by luminescence measurements and further verified by immunoblot analysis. As depicted in Fig. 3B and C, and Fig. S4B, in the absence of calcium ions in the medium about 50% of the BopN^rep^ molecules were secreted and ~ 50% remained intracellular. A slightly lower secretion efficiency of BopN^rep^ was observed in a medium with 0.1 mM Ca^2+^. In contrast, at high Ca^2+^ concentrations of 0.5, 1, and 2 mM, the majority of produced BopN^rep^ molecules were intracellular, indicating a very low secretion efficacy. Interestingly, as also shown in Fig. 3B, deletion of the *bscN* gene encoding for the T3SS ATPase essential for T3SS function, yielded a decrease of intracellular BopN^rep^ level compared to the wild-type strain, likely due to a negative feedback loop on BopN^rep^ transcription/translation and/or stability. Overall, these data demonstrated that BopN secretion is prevented in the secretion-restrictive calcium-rich medium.

**FIG 4:**
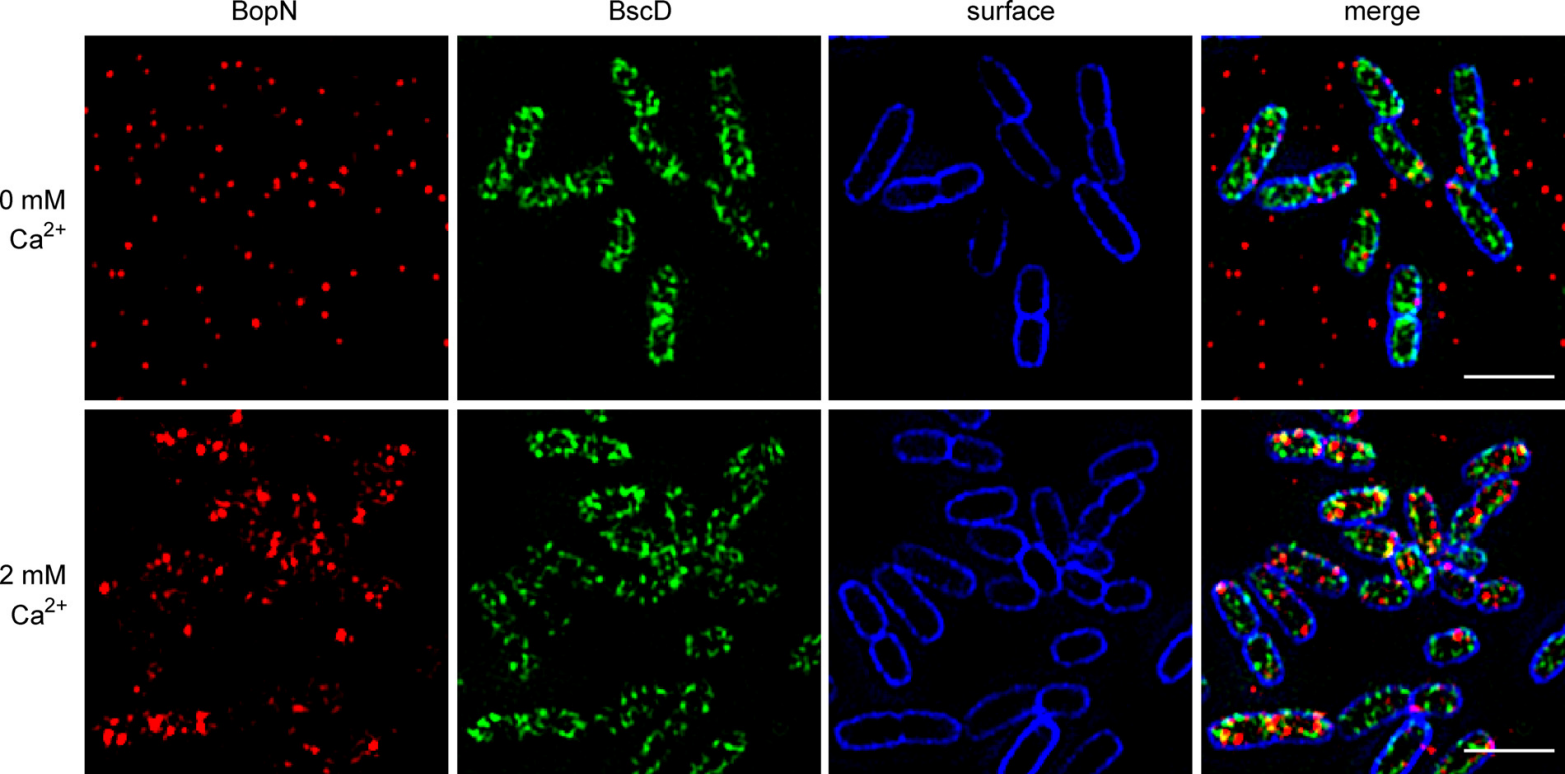
Localizations of BopN protein and the injectisome inner ring component mNG-BscD partially overlap in calcium-rich medium. Cells of *mNG-bscD*/*bopN*^rep^ were fixed on polylysine-coated coverslips after incubation for 90 min in the presence or absence of 2 mM Ca^2+^. BopN^rep^ was visualized with an anti-SPOT nanobody conjugated to ATTO594, while the outer surface of *Bordetella* was stained with a rabbit anti-*Bordetella* serum followed by anti-rabbit IgG-DyLight 405 conjugate. A single focal plane of a Z-stack is shown. Fluorescence images shown are representative of 3 independent experiments. Scale bar represents 2 μm.

We next examined the localization of BopN^rep^ in cells of B. bronchiseptica using structured illumination microscopy (SIM). As revealed by immunofluorescence labeling with an anti-SPOT nanobody conjugated to ATTO594 (Fig. 3D), the BopN^rep^ protein localized to the bacterial periphery under secretion-restrictive conditions (2 mM Ca^2+^). The majority of BopN^rep^ foci were located beneath the bacterial cell envelope and were more concentrated at the poles of the bacterial cells. In contrast, under secretion-permissive conditions (0 mM Ca^2+^) much less intracellular BopN^rep^ foci were detected and their preferential association with cell poles was lost (Fig. 3D and Fig. S4C). To determine whether the distribution of the BopN^rep^ foci under the calcium-rich conditions was consistent with the localization of *Bordetella* injectisomes, we tagged the needle complex inner ring component BscD at its cytoplasmic N-terminus with the mNeonGreen (mNG) fluorescent protein. Importantly, tagging of the BscD protein did not affect injectisome function, as the derived *mNG-bscD*/*bopN*^rep^ strain elicited the same T3SS-dependent cytotoxicity as the wild-type or *bopN*^rep^ reporter strain (Fig. S4A). Furthermore, as shown in Fig. 4, the mNG-BscD fusion protein localized to the cell periphery, like BopN^rep^. Interestingly, only a partial overlap of localization of BopN^rep^ and mNG-BscD proteins was observed. The mNG-BscD foci were more numerous than the BopN^rep^ foci and exhibited a more regular pattern at the cell periphery (Fig. 4). In summary, these results show that under secretion-restrictive culture conditions (2 mM Ca^2+^), BopN is localized to the periphery of *Bordetella* cells and is exported in response to a low calcium concentration, which is an artificial trigger of type III secretion in B. bronchiseptica.

### BopN is injected early upon contact with host cells but does not modulate NF-κB signaling in epithelial cells.

**FIG 5:**
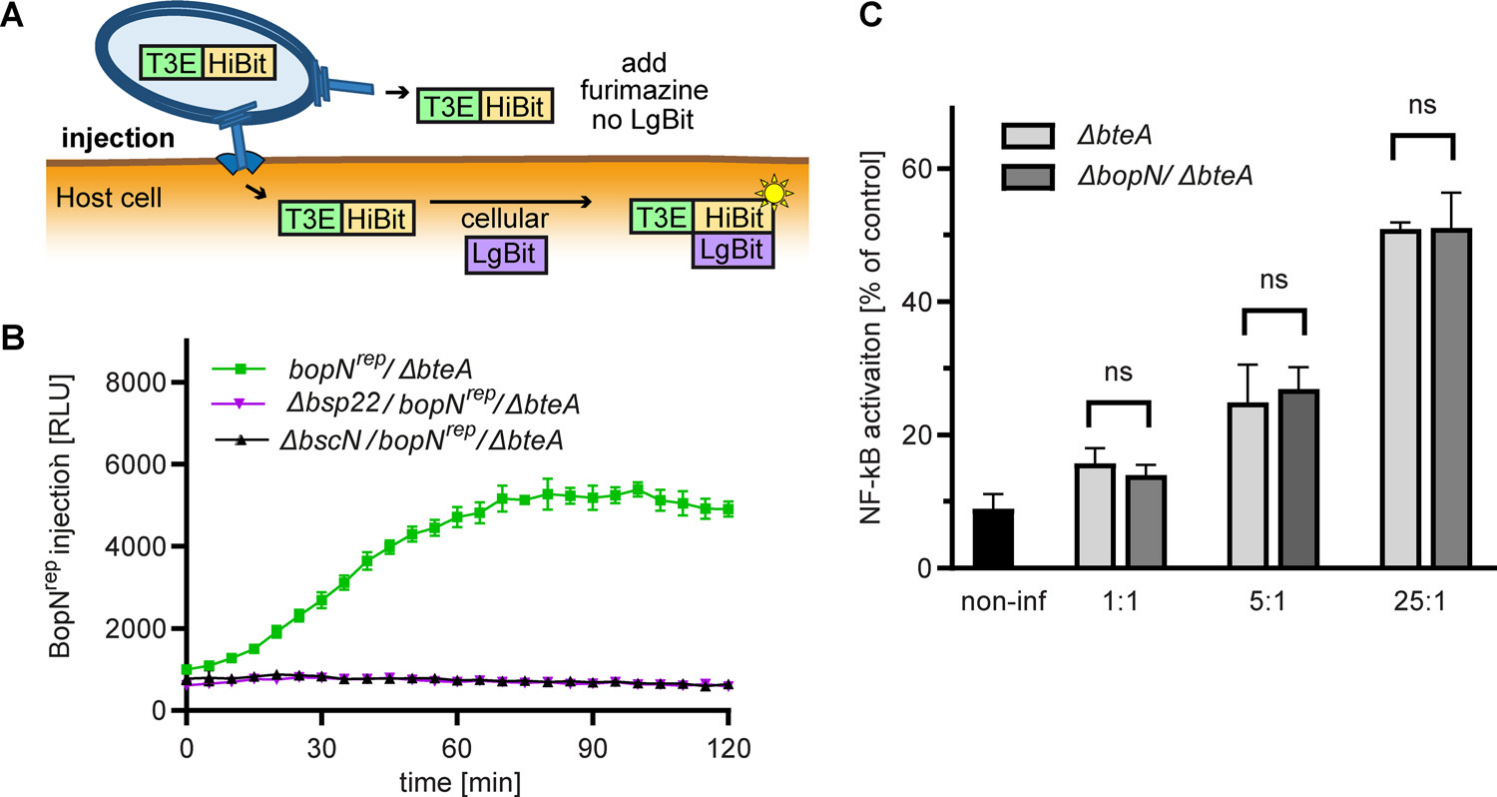
BopN is injected early upon contact with host cells and does not on its own modulate NF-κB signaling in epithelial cells. (A) Schematic representation of HiBit-LgBit functional complementation for real-time monitoring of protein injection. LgBit-expressing host cells are infected in the presence of a cell-permeable furimazine substrate. Injection of the HiBit-fused protein of interest, T3E, is detected as a luminescent signal after complementation with LgBit expressed in the host cell cytosol. The absence of LgBit in the medium surrounding the cells prevents the detection of any amounts of the secreted T3E. (B) BopN is injected early upon contact with host cells. LgBit-expressing HeLa cells were infected with *bopN*^rep^/Δ*bteA* and the indicated derivates at MOI of 5:1. Luminescence measurements were performed at 5-min intervals and were expressed as relative luminescence units (RLU). Shown are mean values ± SD of triplicate wells from a representative experiment of 3 experiments performed. (C) BopN does not modulate NF-κB signaling in epithelial cells. A549 Dual reporter cells encoding secreted embryonic alkaline phosphatase (SEAP) under the control of the NF-κB-responsive promoter were infected at the indicated MOI. The amount of secreted SEAP in the culture medium was determined after 20 h of infection and is expressed as % of SEAP in the culture medium of cells stimulated with 1 ng/mL TNF-α. Values represent the means ± SD from 2 independent experiments (*n* = 4). Statistical analysis was performed using an unpaired two-tailed *t* test; ns, not significant.

To gain further insight into the mode of action of BopN, we next examined its fate upon bacterial contact with host cells. Specifically, we determined the translocation kinetics of BopN^rep^ into host cells using the split-luciferase system. Toward this aim, we generated a nontoxic *bopN*^rep^/Δ*bteA* B. bronchiseptica strain by in-frame deletion of the *bteA* open reading frame and expressed LgBit in the host cell. In this scenario, shown schematically in Fig. 5A, LgBit can be complemented only by the HiBit-tagged BopN^rep^ protein injected into the cell cytosol by the T3SS. In the presence of a cell-permeable furimazine substrate, the formed LgBit/HiBit-tagged BopN^rep^ complex then generates a luminescence signal (48, 49).

Accordingly, as shown in Fig. 5B, no luminescence signal was detected when LgBit-expressing HeLa cells were infected with a Δ*bsp22*-derivative containing a deletion of the open reading frame for the T3SS tip protein (Δ*bsp22*/*bopN*^rep^/Δ*bteA*) or a secretion-deficient Δ*bscN* derivative (Δ*bsN*/*bopN*^rep^/Δ*bteA*) unable of T3SS-mediated injection. In contrast, a steady increase in luminescence was observed for up to 60 min postinfection when LgBit-expressing HeLa cells were infected with the *bopN*^rep^/Δ*bteA* strain at the same multiplicity of infection (MOI) 5:1 (Fig. 5B). These data indicate that a preexisting pool of BopN^rep^ molecules was injected early upon bacterial contact with host cells.

We also investigated whether BopN-injected into host epithelial cells modulates their NF-κB signaling. To this end, human A549 alveolar basal reporter cells, encoding the secreted embryonic alkaline phosphatase (SEAP) under the control of the NF-κB-responsive promoter, were infected with B. bronchiseptica bacteria and SEAP activity was determined in the culture supernatants. We were unable to perform these experiments with wild-type B. bronchiseptica due to acute BteA-induced cytotoxicity and cell lysis. The BteA-secreting wild-type or Δ*bopN* strains of B. bronchiseptica did not elicit any detectable SEAP production at MOI 1:1, 5:1 or 25:1 (data not shown). In contrast, SEAP activity in the supernatant increased with increasing MOI when cells were infected with Δ*bteA* and Δ*bopN*/Δ*bteA* derivatives (Fig. 5C). Importantly, no statistically significant difference (unpaired two-tailed *t* test using the corresponding MOI) was observed between the SEAP activities in the supernatants from Δ*bteA* and Δ*bopN*/Δ*bteA* bacteria-infected cells (Fig. 5C). Overall, these data demonstrate that BopN is injected early upon contact with host cells but does not modulate the NF-κB-signaling in epithelial cells.

### BopN is required for efficient and targeted BteA injection into host cells.

Having shown that BopN controls BteA secretion and is itself injected into host cells early upon bacterial contact with cells, it was important to investigate whether deletion of the *bopN* gene will also result in the loss of control over BteA delivery to host cells. To this end, we analyzed the amounts of BteA^rep^ that leaked into the culture medium and the amounts of BteA^rep^ that were injected into host cells during infection.

**FIG 6:**
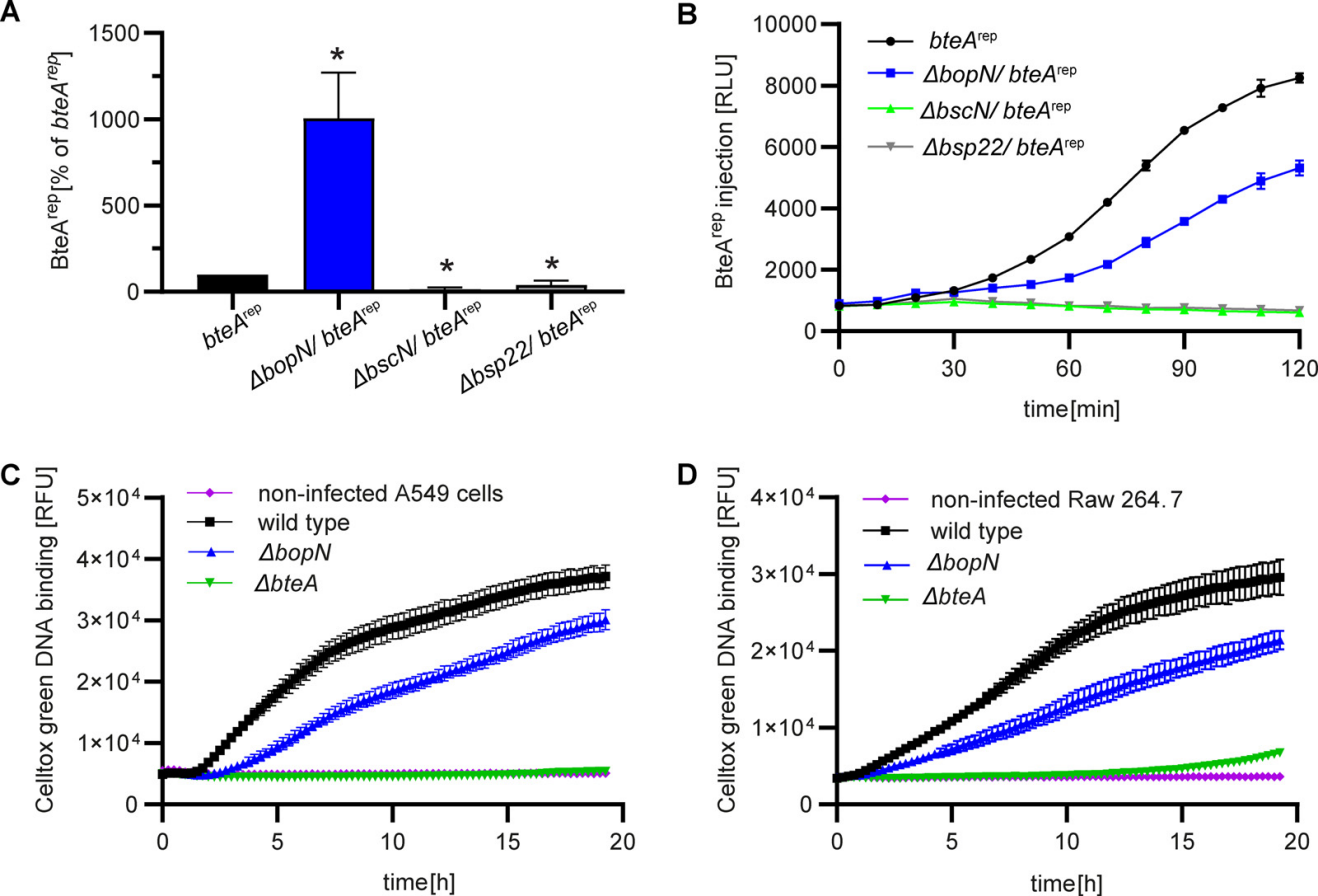
BopN is required for efficient and targeted BteA injection into host cells. (A) BopN controls the leakage of BteA into the surroundings of host cells during infection. HeLa cells were infected with the *bteA*^rep^ and the indicated derivatives at MOI of 5:1. The amount of BteA^rep^ in the medium surrounding the cells was determined by luminescence measurements after 3 h of infection and expressed as % of BteA^rep^ detected in the medium of *bteA*^rep^-infected cells. Values represent the means ± SD from 3 independent experiments (*n* = 3). Significant differences (*, *P < *0.05, unpaired two-tailed *t* test) between BteA^rep^ secreted by derivative and *bteA*^rep^ strains are indicated. (B) Inactivation of BopN leads to defects in the delivery of BteA into host cells. LgBit-expressing HeLa cells were infected with *bteA*^rep^ and the indicated derivates at MOI of 5:1. Luminescence measurements were performed at 5-min intervals and are reported as relative luminescence units (RLU). Shown are mean values ± SD of triplicate wells from a representative experiment. Data are representative of 3 independent experiments. (C) and (D) Inactivation of BopN results in reduced BteA-mediated cytotoxicity. Toxicity of the indicated strains against human A549 alveolar epithelial cells infected at MOI 5:1 (C) and against mouse Raw 264.7 macrophages infected at MOI 1:1 (D) was measured as real-time membrane permeabilization kinetics and monitored by fluorescence of the DNA-binding dye CellTox Green. Shown are mean values ± SD of triplicate wells from a representative experiment. Data are representative of 2 independent experiments.

Leakage of BteA^rep^ into the medium during infection of HeLa cells was quantified as luminescence signal after addition of LgBit protein and furimazine substrate to aliquots of the spent medium. Interestingly, the Δ*bopN* (Δ*bopN*/*bteA*^rep^) bacteria leaked approximately 10-fold higher amounts of BteA^rep^ into culture medium than the wild-type reporter *bteA*^rep^ B. bronchiseptica strain (Fig. 6A). In contrast, such increase was not observed with the T3SS-tip-deficient derivative (Δ*bsp22*/*bteA^rep^*), which exhibited a lower leakage of BteA^rep^ than the wild-type strain. As expected, negligible amounts of leaked BteA^rep^ were detected in infection experiments with the secretion-deficient strain (Δ*bscN*/*bteA*^rep^) (Fig. 6A), confirming that occurrence of the luminescence signal depended on T3SS function. When the same reporter strains were used to quantify the injection of BteA^rep^ into LgBit-expressing HeLa cells by luminescence measurements, the wild-type *bteA*^rep^ strain translocated the largest amounts of BteA^rep^ into the cells (Fig. 6B). The luminescence observed for the Δ*bopN* strain (Δ*bopN*/*bteA*^rep^), reflecting the amount of BteA^rep^ injected into the cells, was reproducibly lower and slightly delayed. As expected, no injection of BteA^rep^ was detected with the translocation-deficient (Δ*bsp22*/*bteA*^rep^) and the secretion-deficient (Δ*bscN*/*bteA*^rep^) strains, respectively (Fig. 6B). Overall, these data suggest that the BopN protein is required for effective and targeted delivery of BteA by *Bordetella* T3SS into host cells.

To confirm these results and indirectly evaluate the efficiency of T3SS-mediated injection of the cytotoxic effector BteA into host cells, we assessed the BteA-induced cytotoxicity toward human A549 alveolar basal epithelial cells and murine Raw 264.7 macrophages. Consistent with previous results, infection with the Δ*bopN* mutant yielded lower cytotoxicity on A549 cells and Raw 264.7 macrophages than infection with the wild-type bacteria (Fig. 6C and D). This confirmed the defect in T3SS-mediated BteA injection into cells in the absence of the BopN protein. Indeed, the observed cytotoxicity of B. bronchiseptica toward host cells was due to the action of the effector BteA, as the Δ*bteA* mutant strain did not cause any early cytotoxicity at the MOIs used for A549 cells (5:1) or Raw 264.7 macrophages (1:1).

In summary, our data show that BopN controls the targeted delivery of BteA into the host cell cytosol and that BopN is the gatekeeper of T3SS in bordetellae.

## DISCUSSION

In this study, we have solved the structure of the BopN protein fragment (residues 83 to 365) at a refinement 1.95 Å and report that it is similar to the structures of previously characterized T3SS gatekeeper proteins of Gram-negative bacteria. We further demonstrate experimentally that BopN is a gatekeeper of the *Bordetella* T3SS.

The T3SS is employed by many bacterial pathogens to translocate effector proteins directly into host cell cytosol to modulate host cell functions to the advantage of the bacteria. While translocated effector proteins and the transcriptional and posttranscriptional network regulating the T3SS expression are unique to each species, the biogenesis of T3SS injectisomes, their structural components and their overall architecture are broadly conserved (51). Biogenesis of the injectisome is a tightly regulated hierarchical process in which the T3SS substrates are secreted in a specific order (22, 23). Effector proteins are secreted only when the T3SS needle tip senses the host cell *in vivo* or in the presence of an artificial trigger (25, 27–29). Here, we demonstrated that the T3SS secretion of B. bronchiseptica is activated by a low concentration of calcium ions, similar to T3SS secretion of *Yersinia* and EPEC (24–26). The standard SS medium used to culture *Bordetella* cells contains a low calcium ion concentration (~ 0.1 mM) and, therefore, artificially activates *Bordetella* T3SS secretion. When the *Bordetella* culture medium contains calcium ions at the 2 mM physiological concentration found in body fluids, secretion of the BteA effector is inhibited (~ 7-fold). However, how the artificial signal or host cell contact is transmitted to trigger BteA secretion remains to be elucidated, and both mechanical and chemical signals need to be considered (52).

One protein that is critical for triggering the secretion of effectors and their targeted delivery into host cells is the T3SS gatekeeper, referred to as SctW. It has been suggested that the gatekeeper interacts with the SctV protein of the T3SS export apparatus, where it supports the loading of translocator-chaperone complexes but blocks the entry of effector-chaperone complexes. Following an activation signal (contact with the host cell and/or an artificial trigger), the gatekeeper is removed to allow the secretion of effector proteins (31, 33).

Although BopN shares a sequence similarity with YopN and other T3SS gatekeepers (Table 2), its function as a gatekeeper in *Bordetella* injectisome has been controversial. First, the deletion of the *bopN* gene does not affect the overall amounts of T3SS-secreted proteins released into the standard low calcium-containing SS medium (10). Second, the inactivation of BopN was reported to impair BteA-mediated cytotoxicity toward rat L2 pulmonary epithelial cells but not toward murine DC2.4 dendritic cells (10, 19). Third, BopN was found to be translocated into host cells and was reported to impair B. bronchiseptica-induced NF-κB signaling (10, 19). Importantly, we show here that the absence of BopN promotes secretion of the BteA effector under the secretion-restrictive conditions when B. bronchiseptica is grown in calcium-rich modified Stainer-Scholte (SSM) medium, which reveals the gatekeeper function of BopN. Further, we also demonstrated that B. bronchiseptica bacteria lacking BopN exhibit approximately 10-fold increased leakage of BteA into the culture medium during cell infection experiments and impair BteA injection into host cells, which yields reduced BteA-mediated cytotoxicity. Hence, although BopN is required for targeted and efficient translocation of BteA into host cells, its inactivation does not entirely prevent the delivery of BteA into host cells. This is most likely because the absence of BopN does not hinder the secretion of the translocators BopB and BopD and the tip filament Bsp22. Thus, like for the YopN/TyeA gatekeeper complex of *Yersinia* spp. (53, 54), BopN is not required for loading the translocator-chaperone complexes into the export apparatus of *Bordetella* injectisome. Indeed, the gatekeeper-mediated control of translocator secretion is more complex and can differ among species. Most gatekeeper mutants, e.g., mutants of InvE of the Salmonella pathogenicity island 1 (SPI-1) (55) and SsaL of the SPI-2 (27, 33), SepL of EPEC (26), VgpA, and VgpB of *Vibrio* (29) exhibit defects in their secretion, and thereby efficiently block effector translocation into host cells. In contrast, the MxiC mutant of *Shigella* does not impair the translocator protein secretion (38) and the YopN/TyeA mutant of *Yersinia* even upregulates their secretion (53, 54).

The here presented functional data are also supported by the solved structure of truncated BopN (residues 83 to 365) that exhibits structural similarity to the characterized gatekeeper proteins belonging to the YopN/InvE/MxiC (or LcrE) family (InterPro entry IPR013401). The BopN structure is rod-shaped and consists of 3 X-bundle domains with 4 α-helices, potentially allowing simultaneous interactions with multiple partner proteins, while having a disordered N-terminus containing the secretion signal and a chaperone-binding region not included in the structure (45, 56). Interestingly, the BopN molecule exhibits a different relative orientation of the 3 X-bundle domains compared to other gatekeepers (Fig. S1), which could reflect the contribution of the missing N-terminal part to the overall structure, a different state of the trapped BopN molecule due to different crystallization conditions, or an actual difference of the BopN molecule. At present, it is not known why some gatekeepers, such as BopN or YopN, are secreted by the injectisome upon activation signal, whereas others, such as SepL, are not secreted. The presence of a secretion signal *per se* does not provide an explanation, as this was revealed also in the non-secreted SepL protein upon truncation of its C-terminal portion (57). Thus, the fate of the gatekeeper more likely depends on its interactions with other components of the injectisome and probably also on the ATPase SctN, which accounts for the ATP-dependent release of chaperones and for the unfolding of the corresponding secreted proteins (58). Indeed, further studies are needed to investigate the interactions of BopN in *Bordetella* T3SS and to uncover the mechanisms of how BopN senses the low calcium concentration and activates the secretion and translocation of BteA. To gain an initial insight into the action of BopN, we analyzed localization of BopN in B. bronchiseptica cells and its distribution compared to T3SS injectisomes. We found that BopN distributed in patches beneath the cell surface. The tagged mNG-BscD, inner ring component of the needle complex, also localized in foci at the cell periphery. These foci were visible in all cells and resembled the foci of SctD from Y. enterocolitica (59, 60). Interestingly, the BopN foci appeared to be located more in the cytoplasm than the BscD foci and were less numerous. Not all BscD foci were occupied by BopN. The number of intracellular BopN foci decreased under secretion-promoting conditions, confirming that BopN is secreted in response to an artificial trigger. We also demonstrated that BopN is released from bacterial cells and translocated into the host cells early after the host cell contact. However, we did not detect any BopN-mediated modulation of NF-κB signaling, contrary to what has been reported previously (19). This may be related to the use of different cells and assays.

**FIG 7:**
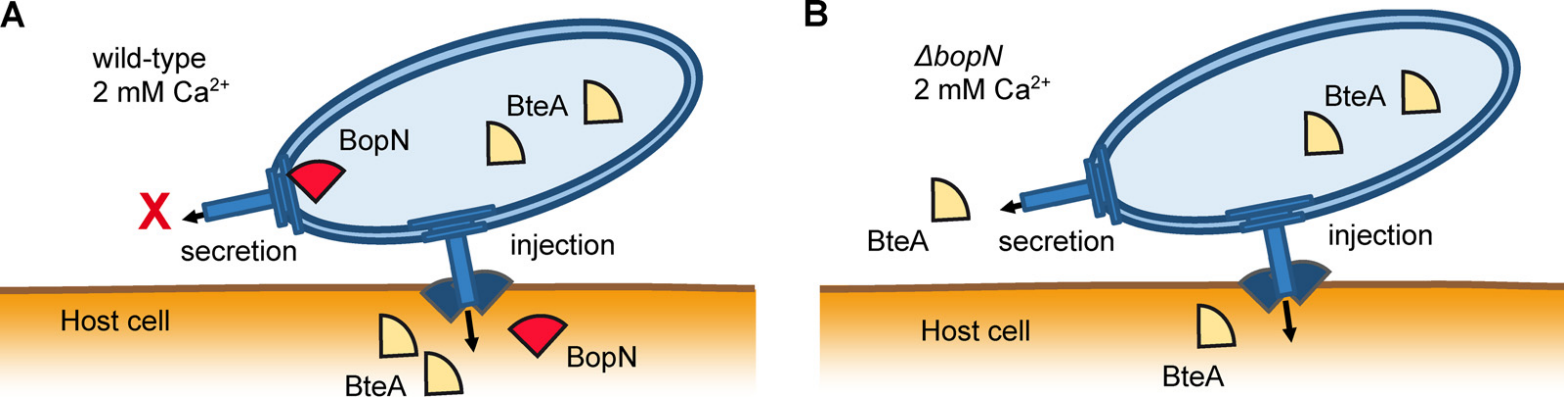
Proposed model for the BopN action in bordetellae. (A) In wild-type cells, BopN prevents secretion of BteA before contact with the host cell. The host cell contact with the injectisome triggers translocation of BopN into the host cell and also allows for targeted translocation of BteA. (B) In the Δ*bopN* mutant, targeted BteA translocation does not take place due to the absence of BopN, therefore, BteA leaks into the surroundings of the infected cells. This results in lower amounts of BteA being injected into the host cells.

Overall, our study provides new insights into the action of the *Bordetella* BopN protein by showing that BopN protein promotes efficient and targeted T3SS-mediated injection of BteA into host cells and acts as the T3SS gatekeeper, as summarized by the model in Fig. 7. These findings are important for understanding the mechanism of action of the T3SS of *Bordetella* and for future deciphering of the role of its effector(s) in *Bordetella* infections.

## MATERIALS AND METHODS

### Bacterial strains and growth conditions.

The bacterial strains used in this study are listed in Table S1. E. coli strain XL1-Blue was used for plasmid construction, E. coli strain SM10λ pir was used for plasmid transfer into B. bronchiseptica RB50 by bacterial conjugation, and E. coli BL21 λ(DE3) and XL-1 Blue were employed for expression of recombinant proteins. E. coli strains were cultivated at 37°C in LB agar or LB broth. When appropriate, the LB medium was supplemented with 100 μg/mL of ampicillin or 60 μg/mL of kanamycin. The parental B. bronchiseptica RB50 and derived mutant strains were grown on Bordet-Gengou (BG) agar medium (Difco) supplemented with 1% glycerol and 15% defibrinated sheep blood (LabMediaServis) at 37°C and 5% CO_2_ for 48 h. Liquid cultures of *Bordetella* strains were performed in modified Stainer-Scholte (SSM) medium (61) supplemented with 5 g/l of Casamino Acids (Difco) at 37°C. To maximize the expression of the type III secretion system for assays, SSM medium was formulated with reduced l-glutamate (monosodium salt) concentration (11.5 mM, 2.14 g/l) and no FeSO_4_.7H_2_O added (62, 63). To obtain the indicated concentration of calcium ions within SSM, 1 M CaCl_2_ was used. B. bronchiseptica for assays and inoculations was grown to a mid-exponential phase (OD_600_ 1.5) in calcium-rich SSM medium (2 mM Ca^2+^) unless otherwise indicated.

### Cell culture and generation of LgBit-expressing HeLa cells.

Dulbecco’s Modified Eagle Medium ([DMEM] Sigma) supplemented with 10% fetal bovine serum (DMEM-10% [FBS]), was used to cultivate the following cell lines at 37°C and 5% CO_2_: HeLa (ATCC CCL-2, human cervical adenocarcinoma), LgBit-expressing HeLa (see below), 293T (ATCC CRL-3216, human epithelial kidney cell line), A549 (ATCC CCL-185, human lung carcinoma epithelial cells), A549-Dual (InvivoGen a549d-nfis, A549 cell reporter derivatives encoding secreted embryonic alkaline phosphatase [SEAP] under the control of the IFN-β minimal promoter fused to five NF-κB binding sites) and RAW 264.7 macrophages (ATCC TIB 71, Abelson leukemia virus-transformed murine cell line). LgBit-expressing HeLa cells were generated by lentiviral transduction of the parental HeLa cell line. The coding sequence of LgBit was subcloned into a modified pLJM1-EGFP vector (Addgene #19319) in-frame with N-terminal FLAG epitope. VSV-pseudotyped viruses were then produced by co-transfection of 6 μg pLJM1-FLAG-LgBit, 6 μg pCMV-VSV-G, and 6 μg psPAX2 plasmids using Lipofectame 2000 (Invitrogen) into 293T cells grown in a 10-cm dish. The cell culture supernatant was collected 48 h after transfection and used to transduce the parental HeLa cells in the presence of Polybrene (8 μg/mL). Twenty-four hours after transduction, cells were split, and transduced cells were selected by puromycin (0.5 μg/mL). Expression of flag-LgBit was verified by immunoblotting with the anti-flag M2 antibody (Sigma-Aldrich).

### Plasmid construction and *Bordetella* allelic exchange.

Plasmids used in this study are listed in Table S2, and were constructed using the T4 DNA ligase or Gibson assembly strategy (64). PCR amplifications were performed from chromosomal DNA of B. pertussis Tohama I or B. bronchiseptica RB50 using Herculase II Phusion DNA polymerase (Agilent). The LgBit coding sequence was synthesized as GeneArt strings fragments (Invitrogen, Thermo Fisher Scientific), and mNeonGreen coding sequence was amplified from mNeonGreen-Rab7 vector (Addgene #129603). All constructs were verified by DNA sequencing (Eurofins Genomics). Mutant B. bronchiseptica strains were constructed by homologous recombination using the suicide allelic exchange vector pSS4245 as previously described (18). The presence of the introduced mutations was confirmed by PCR amplification of the relevant regions of the *Bordetella* chromosome, followed by agarose gel analysis and DNA sequencing (Eurofins Genomics).

### BopN protein production and purification.

The recombinant 6×His-tagged BopN protein was produced in the E. coli BL21 λ(DE3) cells transformed with the pET28b-*Bp*TohamaI-BopN plasmid construct. The bacterial cells were grown in LB broth at 37°C supplemented with 60 μg/mL kanamycin. Expression of the protein was induced by the addition of 0.5 mM isopropyl 1-thio-β-d-galactopyranoside (IPTG, Alexis) when the optical density at 600 nm reached 0.6 and cultures were further grown at 37°C for additional 4 h. The cells were washed in TN buffer (50 mM Tris-HCl, pH 8.0, 150 mM NaCl) and disrupted by ultrasonic processor (Misonix S-4000, Misonix). The cell lysate was centrifuged at 40,000 g at 4°C for 30 min and the supernatant was loaded on a Ni Sepharose 6 High Performance column (Cytiva) equilibrated with TN buffer. The column was extensively washed with TN buffer supplemented with 50 mM imidazole and the BopN protein was eluted with TN buffer supplemented with 200 mM imidazole. The protein fractions were pooled and dialyzed against 50 mM Tris-HCl (pH 8), 150 mM NaCl at 4°C for 16 h. The dialyzed BopN protein was diluted with TN buffer to a concentration of 1 mg/mL and incubated with trypsin (Sigma) at molar ratio of 1:1,000. After 1 h of incubation at 25°C, the mixture was loaded on Ni Sepharose 6 High Performance column (Cytiva) equilibrated with TN buffer. The flow-through fractions containing the truncated BopN protein (BopN_69-365_) were concentrated by ultrafiltration using a 10-kDa cutoff membrane (Amicon) and loaded on a Superdex 75 HR gel filtration column (GE Healthcare) equilibrated with 10 mM Tris-HCl (pH 7.4) and 150 mM NaCl. The eluted proteins were concentrated by ultrafiltration and stored at 4°C for further use. The purity of protein samples was monitored by SDS-PAGE. Protein concentrations were determined by Bradford assay (Bio-Rad) using bovine serum albumin (Sigma) as a standard.

### Crystallization of the BopN protein.

Crystallization screening was performed in a sitting-drop vapour-diffusion setup using a Gryphon (Art Robbins Instruments) and Oryx4 (Douglas Instruments Ltd) crystallization robots in MRC 2 Well (Hampton Research [HR]) and Combi Clover Junior crystallization plates (Rigaku Reagents), respectively. The diffraction quality BopN crystals with the dimensions of 350 × 50 × 30 μm grew within a week at 4°C in 0.16 M calcium acetate, 0.08 M sodium cacodylate, 14.4% (wt/vol) PEG 8000, 20% (vol/vol) glycerol employing a protein stock concentration of 8 mg/mL and protein-to-precipitant ratio 2:1.

### Data collection and processing.

Diffraction data were collected from an individual crystal at 100 K using synchrotron radiation on the MX14.1 beamline at BESSY II synchrotron (Helmholtz-Zentrum) equipped with the Pilatus3 S 2M detector (Dectris). 1800 diffraction images were collected with 0.1 oscillation angle. Collected data were processed using XDS (65) in XDSAPP interface (66).

### Structure solution and refinement.

The search model was generated by ARCIMBOLDO light (67, 68), based on the combination of locating small model fragments with density modification with the program SHELXE (69). The protein structure was solved by automated model-building method BUCCANEER (70). The structure was refined by REFMAC5 (71) and manually modeled using COOT (72). MolProbity server (73) was used for final model geometry validation. For the determination of the protein assembly, PDBePISA (74) was applied. The complete information about the data collection and refinement statistics is provided in Table 1. Coordinates and structure factors for the BopN protein were deposited in the Protein Data Bank under the accession code 7YYG.

### Determination of intracellular and secreted BopN^rep^ and BteA^rep^ levels by luminescence measurements.

To determine calcium-induced BopN secretion, *bopN*^rep^ reporter bacteria were grown to exponential phase (OD_600_ = 1.2) in calcium-rich SSM (2 mM Ca^2+^) and transferred into SSM medium with different concentrations of Ca^2+^. Following incubation for 90 min at 37°C, the cultures were centrifuged (10 min; 14,000 g) and amounts of BopN^rep^ in supernatants (secreted BopN^rep^) and extracts of cell pellets (intracellular BopN^rep^) were determined (see below). To prepare the extracts, pellets were resuspended in 50 mM Tris-HCl, pH 8.0, and the cells were disrupted in 2 beating cycles of 3 min with 0.1 mm glass beads (Scientific Industries) using the Disruptor Genie (Scientific Industries). The extracts were clarified by centrifugation (10 min; 14,000 g).

For analysis of secreted and intracellular BteA amounts, reporter bacteria of *bteA*^rep^ grown to exponential phase (OD_600_ = 1.2) in calcium-rich SSM (2 mM Ca^2+^) were inoculated to an OD_600_ of 0.15 into 50 mL of SSM medium with or without 2 mM Ca^2+^ and grown for various times at 37°C. One-mL aliquots of the cultures were taken after 3 h, 6 h, 9 h, and 24 h of growth. No difference in OD_600_ was observed between cultures in SSM with or without 2 mM Ca^2+^. After aliquot centrifugation (10 min; 14,000 g), amounts of BteA^rep^ in supernatants and cell extracts were assessed with Nano-Glo HiBit system.

Nano-Glo HiBiT Extracellular Detection System (Cat. No. N2420, Promega) was used, according to manufacturer's instructions. Briefly, supernatants and cell extracts were mixed with recombinant LgBit protein and furimazine substrate in Nano-Glo buffer, and lluminescence was measured using the FLUOstar Omega microplate reader (BMG LABTECH) or the TecanSpark microplate reader (Tecan). The purified recombinant protein BopN^rep^ and BteA^rep^ were used for the calibration curve and calculation of protein amounts.

### Production and purification of recombinant BopN^rep^ and BteA^rep^.

The recombinant GST-tagged BopN^rep^ (aa 83 to 365 of BopN with C-terminal HiBit-3xFLAG-SPOT peptide) and BteA^rep^ (aa 1 to 130 of BteA with C-terminal HiBit peptide) were produced in E. coli XL-1 from pGEX-6P1 expression vector (GE Healthcare). Exponential E. coli cultures grown at 30°C were induced for protein production by adding IPTG to 0.1 mM at OD_600_ = 0.3 and grown for an additional 16 h at 25°C. Bacterial cells were harvested by centrifugation, and the cell pellet was resuspended in ice-cold 50 mM Tris-HCl pH 7.4, 150 mM NaCl, and Complete Mini protease inhibitors (EDTA free, Roche). Bacterial cells were disrupted by ultrasound, and the lysate was clarified by centrifugation (20,000 g, 30 min). The recombinant proteins were purified from the supernatant fraction using columns prepacked with Glutathione-Sepharose 4B (Amersham). The resin with bound proteins was washed with 50 mM Tris-HCl pH 7.4, 150 mM NaCl and proteins were eluted with 10 mM reduced glutathione in 50 mM Tris-HCl pH 7.4, 150 mM NaCl. Protein preparations were dialyzed overnight into 50 mM Tris-HCl pH 7.4 and 150 mM NaCl. The integrity and purity of recombinant proteins were verified by SDS-PAGE electrophoresis followed by Coomassie blue staining, and protein concentration was determined by Bradford assay (Bio-Rad) using bovine serum albumin (Sigma) as a standard.

### Immunofluorescence staining and structured illumination microscopy.

Following centrifugation (5 min; 8,000 g) to remove SSM medium containing 2 mM Ca^2+^, bacteria were re-inoculated into SSM medium with or without 2 mM Ca^2+^. After incubation for 90 min at 37°C, cultures were spotted onto high-precision coverslips (#1.5 H), which were cleaned in 1N HCl and coated with 0.01% poly-L-lysine before use. Bacteria were allowed to adhere for 30 min and then fixed with 4% PFA in phosphate-buffered saline (PBS) for 75 min at RT. After washing with PBS, cells were permeabilized with 0.2% Triton X-100 in PBS at RT for 45 min and then washed in PBS with 0.05% Tween 20 (PBST). Blocking was performed with 4% BSA in PBST for 1 h, and primary antibodies were applied in 1% BSA in PBST at the following dilutions: SPOT-Label ATTO594 (Chromotek) at 1:600, and rabbit anti-B. pertussis serum (generously provided by Dr. Vecerek, Institute of Microbiology, Prague, Czech Republic) at 1:1,000. After incubation for 2 h at 37°C and washing with PBST (3 × 5 min), the secondary goat anti-rabbit antibody labeled with DyLight 405 (Jackson Immunoresearch) was applied in 1% BSA in PBST at the 1: 600 dilution. After incubation for 30 min at 37°C, Vectashield (H-1000-10, Vector Laboratories) was used to mount the coverslips containing the samples onto glass slides.

The DeltaVision OMX imaging platform was used to acquire images with 3D structured illumination microscopy (SIM). The system was equipped with the PLAN APO N 60x oil objective, N.A. 1.42; FWD 0.15; CG 0, four PCO and an Edge 5.5 sCMOS camera (readout speeds 95 MHz, 286 Mhz, 15 bit, pixel size: 6.5 μm). For excitation of fluorescent proteins and the labels on the antibodies, 405 nm diode, 488 nm OPSL, and 564 nm OPSL were used in combination with emission filters 435.5/31, 528/48, and 609/37 nm. SoftWoRx software (Applied Precision) was used for image reconstruction, deconvolution, and registration. Image processing, which consisted of cropping and brightness/contrast adjustment, was performed in FIJI ([75], NIH Bethesda), and final images were assembled in Adobe Illustrator (Adobe).

### Analysis of intracellular and secreted proteins by immunoblotting.

Bacteria of *bteA*^rep^ strains were grown in the SSM medium with and without 2 mM Ca^2+^ overnight at 37°C. Alternatively, *bopN^rep^* bacteria grown in calcium-rich medium (2 mM Ca^2+^) were transferred into medium with various Ca^2+^ concentrations (0, 0.1, 0.5, 1, and 2 mM) and incubated for 90 min. For analysis of intracellular protein content, bacterial cultures were centrifuged (30 min; 30,000 g) and pellets were lysed in 8M urea and 50 mM Tris-HCl, pH 8.0. Protein extracts were clarified by centrifugation (5 min; 14,000 g) and mixed with SDS-PAGE sample loading buffer. To analyze secreted protein content, culture supernatants were precipitated with 10% trichloroacetic acid overnight at 4°C, washed with acetone, dissolved in 8M urea, 50 mM Tris-HCl, pH 8.0, and mixed with SDS-PAGE sample loading buffer. Samples with ODs equivalent to 0.1 OD_600_ unit (whole-cell lysates) or 1 OD_600_ unit (bacterial supernatants) were separated by SDS-PAGE electrophoresis and transferred onto a nitrocellulose membrane. Membranes were probed overnight with mouse polyclonal antibodies raised against BopN (dilution 1:10,000), Bsp22 (dilution 1:10,000), BopD (dilution 1:10,000), or BopB (dilution 1:10,000), all kindly provided by Branislav Vecerek, Institute of Microbiology, Prague, Czech Republic. The detected proteins were revealed with 1:3,000-diluted horseradish peroxidase (HRP)-conjugated anti-mouse IgG secondary antibodies (GE Healthcare) using a Pierce ECL chemiluminescence substrate (Thermo Fisher Scientific) and an Image Quant LAS 4000 station (GE Healthcare).

### Determination of leakage of BteA^rep^ during infection.

To assess the amount of BteA^rep^ that leaked into the medium during cell infection, HeLa cells (1 × 10^6^ per well) in DMEM-10% FBS were seeded in a 12-well plate and allowed to adhere overnight. Bacteria of *bteA*^rep^ strains grown in calcium-rich SSM (2 mM Ca^2+^) were washed in DMEM-10% FBS by centrifugation (5 min; 8,000 g) and added at MOI 5:1. The plate was centrifuged (5 min, 120 g) to allow efficient infection. Three hours after infection at 37°C and 5% CO_2_, aliquots of the medium were collected and clarified by centrifugation at 14,000 g for 10 min. The Nano-Glo HiBiT Extracellular Detection System (Cat.No. N2420, Promega) was used to determine the amount of BteA^rep^ in the aliquots. Purified recombinant BteA^rep^ protein was used for calibration curve and calculation of protein amounts.

### Determination of cellular injection of BopN^rep^ and BteA^rep^.

To assess the cellular injection of BopN^rep^ and BteA^rep^, LgBit-expressing HeLa cells (5 × 10^4^ per well) in DMEM-10% FBS were seeded into 96-well white/clear bottom plate (Corning) and allowed to adhere overnight. The Nano-Glo Live Cell Assay System (Cat.No. N2011, Promega) was employed to determine the amounts of injected BopN^rep^ or BteA^rep^, according to (48). Bacteria of *bteA*^rep^ and *bopN*^rep^ strains grown in calcium-rich SSM (2 mM Ca^2+^) were washed in DMEM-10% FBS by centrifugation (5 min; 8,000 g) and added at MOI 5:1 along with cell-permeable luciferase substrate and Nano-Glo buffer. After centrifugation (5 min, 120 g), the plate was placed inside the chamber of TecanSpark microplate reader (Tecan) with 37°C and 5% CO_2_ and luminescence measurements were performed for 2 h at 5 min intervals.

### Determination of NF-κB activation.

To assess the BopN-mediated modulation of the NF-κB pathway, A549 Dual reporter cells encoding secreted embryonic alkaline phosphatase (SEAP) under the control of the IFN-β minimal promoter fused to five NF-κB binding sites were used. Cells (2 × 10^4^ per well) in DMEM-10% FBS were seeded in 96-well plates and allowed to attach overnight. The derived mutant strains of B. bronchiseptica RB50 were added at the indicated MOI and centrifuged (5 min, 120 g). After incubation at 37°C and 5% CO_2_ for 20 h, the amount of SEAP in cell culture supernatants was determined using QUANTI-Blue detection reagent (rep-qbs, Invivogen) according to the manufacturer's instructions. The amount of SEAP in the supernatant of cells stimulated with 1 ng/mL TNF-α was taken as 100%.

### Cytotoxicity assay.

Cytotoxicity of B. bronchiseptica toward A549 cells and Raw 264.7 macrophages was determined as changes in cell membrane integrity using the fluorescent DNA-binding dye CellTox Green (Cat. No. G8743, Promega). In brief, 2 × 10^4^ A549 cells or 2.5 × 10^4^ Raw 264.7 macrophages per well were seeded in a 96-well black/clear bottom plate (Corning) in DMEM-10% FBS and allowed to adhere overnight. B. bronchiseptica and derived mutant strains were added at the indicated MOI along with CellTox Green. The plate was then centrifuged (5 min, 120 g) and placed inside the chamber with 37°C and 5% CO*_2_* of the TecanSpark microplate reader (Tecan). Fluorescence measurements at 490ex/525em were performed at 15-min intervals for 20 h.

### Statistical analysis.

The significance of the differences between groups was determined by unpaired two-tailed *t* test. Differences were considered statistically significant at *P < *0.01.

## Supplementary Material

Reviewer comments

## References

[1] Berbers G, van Gageldonk P, van de Kassteele J, Wiedermann U, Desombere I, Dalby T, Toubiana J, Tsiodras S, Ferencz IP, Mullan K, Griskevicius A, Kolupajeva T, Vestrheim DF, Palminha P, Popovici O, Wehlin L, Kastrin T, Maďarová L, Campbell H, Ködmön C, Bacci S, Barkoff A-M, He Q, Serosurveillance Study T. 2021. Circulation of pertussis and poor protection against diphtheria among middle-aged adults in 18 European countries. Nat Commun 12:2871. doi:10.1038/s41467-021-23114-y. 34001895 PMC8128873

[2] Goodnow RA. 1980. Biology of *Bordetella bronchiseptica*. Microbiol Rev 44:722–738. doi:10.1128/mr.44.4.722-738.1980. 7010115 PMC373201

[3] Mattoo S, Cherry JD. 2005. Molecular pathogenesis, epidemiology, and clinical manifestations of respiratory infections due to *Bordetella pertussis* and other *Bordetella* subspecies. Clin Microbiol Rev 18:326–382. doi:10.1128/CMR.18.2.326-382.2005. 15831828 PMC1082800

[4] Parkhill J, Sebaihia M, Preston A, Murphy LD, Thomson N, Harris DE, Holden MTG, Churcher CM, Bentley SD, Mungall KL, Cerdeño-Tárraga AM, Temple L, James K, Harris B, Quail MA, Achtman M, Atkin R, Baker S, Basham D, Bason N, Cherevach I, Chillingworth T, Collins M, Cronin A, Davis P, Doggett J, Feltwell T, Goble A, Hamlin N, Hauser H, Holroyd S, Jagels K, Leather S, Moule S, Norberczak H, O'Neil S, Ormond D, Price C, Rabbinowitsch E, Rutter S, Sanders M, Saunders D, Seeger K, Sharp S, Simmonds M, Skelton J, Squares R, Squares S, Stevens K, Unwin L, et al. 2003. Comparative analysis of the genome sequences of *Bordetella pertussis*, *Bordetella parapertussis* and *Bordetella bronchiseptica*. Nat Genet 35:32–40. doi:10.1038/ng1227. 12910271

[5] Yuk MH, Harvill ET, Miller JF. 1998. The BvgAS virulence control system regulates type III secretion in *Bordetella bronchiseptica*. Mol Microbiol 28:945–959. doi:10.1046/j.1365-2958.1998.00850.x. 9663681

[6] Yuk MH, Harvill ET, Cotter PA, Miller JF. 2000. Modulation of host immune responses, induction of apoptosis and inhibition of NF-kappaB activation by the *Bordetella* type III secretion system. Mol Microbiol 35:991–1004. doi:10.1046/j.1365-2958.2000.01785.x. 10712682

[7] Nicholson TL, Brockmeier SL, Loving CL, Register KB, Kehrli ME, Jr, Shore SM. 2014. The *Bordetella bronchiseptica* type III secretion system is required for persistence and disease severity but not transmission in swine. Infect Immun 82:1092–1103. doi:10.1128/IAI.01115-13. 24366249 PMC3957984

[8] Kamanova J. 2020. *Bordetella* type III secretion injectosome and effector proteins. Front Cell Infect Microbiol 10:466. doi:10.3389/fcimb.2020.00466. 33014891 PMC7498569

[9] Panina EM, Mattoo S, Griffith N, Kozak NA, Yuk MH, Miller JF. 2005. A genome-wide screen identifies a *Bordetella* type III secretion effector and candidate effectors in other species. Mol Microbiol 58:267–279. doi:10.1111/j.1365-2958.2005.04823.x. 16164564

[10] Abe A, Nishimura R, Kuwae A. 2017. *Bordetella* effector BopN is translocated into host cells via its N-terminal residues. Microbiol Immunol 61:206–214. doi:10.1111/1348-0421.12489. 28500733

[11] Kuwae A, Ohishi M, Watanabe M, Nagai M, Abe A. 2003. BopB is a type III secreted protein in *Bordetella bronchiseptica* and is required for cytotoxicity against cultured mammalian cells. Cell Microbiol 5:973–983. doi:10.1046/j.1462-5822.2003.00341.x. 14641181

[12] Nogawa H, Kuwae A, Matsuzawa T, Abe A. 2004. The type III secreted protein BopD in *Bordetella bronchiseptica* is complexed with BopB for pore formation on the host plasma membrane. J Bacteriol 186:3806–3813. doi:10.1128/JB.186.12.3806-3813.2004. 15175294 PMC419950

[13] Medhekar B, Shrivastava R, Mattoo S, Gingery M, Miller JF. 2009. *Bordetella* Bsp22 forms a filamentous type III secretion system tip complex and is immunoprotective *in vitro* and *in vivo*. Mol Microbiol 71:492–504. doi:10.1111/j.1365-2958.2008.06543.x. 19040642 PMC2826148

[14] Kuwae A, Matsuzawa T, Ishikawa N, Abe H, Nonaka T, Fukuda H, Imajoh-Ohmi S, Abe A. 2006. BopC is a novel type III effector secreted by *Bordetella bronchiseptica* and has a critical role in type III-dependent necrotic cell death. J Biol Chem 281:6589–6600. doi:10.1074/jbc.M512711200. 16407269

[15] Stockbauer KE, Foreman-Wykert AK, Miller JF. 2003. *Bordetella* type III secretion induces caspase 1-independent necrosis. Cell Microbiol 5:123–132. doi:10.1046/j.1462-5822.2003.00260.x. 12580948

[16] French CT, Panina EM, Yeh SH, Griffith N, Arambula DG, Miller JF. 2009. The *Bordetella* type III secretion system effector BteA contains a conserved N-terminal motif that guides bacterial virulence factors to lipid rafts. Cell Microbiol 11:1735–1749. doi:10.1111/j.1462-5822.2009.01361.x. 19650828 PMC2788067

[17] Malcova I, Bumba L, Uljanic F, Kuzmenko D, Nedomova J, Kamanova J. 2021. Lipid binding by the N-terminal motif mediates plasma membrane localization of *Bordetella* effector protein BteA. J Biol Chem 296:100607. doi:10.1016/j.jbc.2021.100607. 33789161 PMC8100071

[18] Bayram J, Malcova I, Sinkovec L, Holubova J, Streparola G, Jurnecka D, Kucera J, Sedlacek R, Sebo P, Kamanova J. 2020. Cytotoxicity of the effector protein BteA was attenuated in *Bordetella pertussis* by insertion of an alanine residue. PLoS Pathog 16:e1008512. doi:10.1371/journal.ppat.1008512. 32776984 PMC7446853

[19] Nagamatsu K, Kuwae A, Konaka T, Nagai S, Yoshida S, Eguchi M, Watanabe M, Mimuro H, Koyasu S, Abe A. 2009. *Bordetella* evades the host immune system by inducing IL-10 through a type III effector, BopN. J Exp Med 206:3073–3088. doi:10.1084/jem.20090494. 20008527 PMC2806459

[20] Kerr JR, Rigg GP, Matthews RC, Burnie JP. 1999. The Bpel locus encodes type III secretion machinery in *Bordetella pertussis*. Microb Pathog 27:349–367. doi:10.1006/mpat.1999.0307. 10588908

[21] Fauconnier A, Veithen A, Gueirard P, Antoine R, Wacheul L, Locht C, Bollen A, Godfroid E. 2001. Characterization of the type III secretion locus of *Bordetella pertussis*. Int J Med Microbiol 290:693–705. doi:10.1016/S1438-4221(01)80009-6. 11310448

[22] Diepold A, Wagner S. 2014. Assembly of the bacterial type III secretion machinery. FEMS Microbiol Rev 38:802–822. doi:10.1111/1574-6976.12061. 24484471

[23] Portaliou AG, Tsolis KC, Loos MS, Zorzini V, Economou A. 2016. Type III secretion: building and operating a remarkable nanomachine. Trends Biochem Sci 41:175–189. doi:10.1016/j.tibs.2015.09.005. 26520801

[24] Forsberg A, Bolin I, Norlander L, Wolf-Watz H. 1987. Molecular cloning and expression of calcium-regulated, plasmid-coded proteins of *Y. pseudotuberculosis*. Microb Pathog 2:123–137. doi:10.1016/0882-4010(87)90104-5. 3507554

[25] Lee VT, Mazmanian SK, Schneewind O. 2001. A program of *Yersinia enterocolitica* type III secretion reactions is activated by specific signals. J Bacteriol 183:4970–4978. doi:10.1128/JB.183.17.4970-4978.2001. 11489848 PMC95371

[26] Deng W, Li Y, Hardwidge PR, Frey EA, Pfuetzner RA, Lee S, Gruenheid S, Strynakda NC, Puente JL, Finlay BB. 2005. Regulation of type III secretion hierarchy of translocators and effectors in attaching and effacing bacterial pathogens. Infect Immun 73:2135–2146. doi:10.1128/IAI.73.4.2135-2146.2005. 15784556 PMC1087438

[27] Yu XJ, McGourty K, Liu M, Unsworth KE, Holden DW. 2010. pH sensing by intracellular *Salmonella* induces effector translocation. Science 328:1040–1043. doi:10.1126/science.1189000. 20395475 PMC6485629

[28] Bahrani FK, Sansonetti PJ, Parsot C. 1997. Secretion of Ipa proteins by *Shigella flexneri*: inducer molecules and kinetics of activation. Infect Immun 65:4005–4010. doi:10.1128/iai.65.10.4005-4010.1997. 9316999 PMC175575

[29] Tandhavanant S, Matsuda S, Hiyoshi H, Iida T, Kodama T. 2018. *Vibrio parahaemolyticus* senses intracellular K(+) to translocate type III secretion system 2 effectors effectively. mBio 9:e01366-18. doi:10.1128/mBio.01366-18. PMC6058294 30042203

[30] Shen DK, Blocker AJ. 2016. MxiA, MxiC and IpaD regulate substrate selection and secretion mode in the T3SS of *Shigella flexneri*. PLoS One 11:e0155141. doi:10.1371/journal.pone.0155141. 27171191 PMC4865121

[31] Portaliou AG, Tsolis KC, Loos MS, Balabanidou V, Rayo J, Tsirigotaki A, Crepin VF, Frankel G, Kalodimos CG, Karamanou S, Economou A. 2017. Hierarchical protein targeting and secretion is controlled by an affinity switch in the type III secretion system of enteropathogenic *Escherichia coli*. EMBO J 36:3517–3531. doi:10.15252/embj.201797515. 29109154 PMC5709732

[32] Gaytan MO, Monjaras Feria J, Soto E, Espinosa N, Benitez JM, Georgellis D, Gonzalez-Pedrajo B. 2018. Novel insights into the mechanism of SepL-mediated control of effector secretion in enteropathogenic *Escherichia coli*. Microbiologyopen 7:e00571. doi:10.1002/mbo3.571. 29277965 PMC6011996

[33] Yu XJ, Grabe GJ, Liu M, Mota LJ, Holden DW. 2018. SsaV Interacts with SsaL to control the translocon-to-effector switch in the *Salmonella* SPI-2 type three secretion system. mBio 9:e01149-18. doi:10.1128/mBio.01149-18. 30279280 PMC6168863

[34] Cheng LW, Kay O, Schneewind O. 2001. Regulated secretion of YopN by the type III machinery of *Yersinia enterocolitica*. J Bacteriol 183:5293–5301. doi:10.1128/JB.183.18.5293-5301.2001. 11514512 PMC95411

[35] Boland A, Sory MP, Iriarte M, Kerbourch C, Wattiau P, Cornelis GR. 1996. Status of YopM and YopN in the *Yersinia* Yop virulon: YopM of *Y.enterocolitica* is internalized inside the cytosol of PU5-1.8 macrophages by the YopB, D, N delivery apparatus. EMBO J 15:5191–5201. doi:10.1002/j.1460-2075.1996.tb00904.x. 8895564 PMC452263

[36] Bamyaci S, Ekestubbe S, Nordfelth R, Erttmann SF, Edgren T, Forsberg A. 2018. YopN Is required for efficient effector translocation and virulence in *Yersinia pseudotuberculosis*. Infect Immun 86:e00957-17. doi:10.1128/IAI.00957-17. 29760214 PMC6056859

[37] Rosqvist R, Magnusson KE, Wolf-Watz H. 1994. Target cell contact triggers expression and polarized transfer of *Yersinia* YopE cytotoxin into mammalian cells. EMBO J 13:964–972. doi:10.1002/j.1460-2075.1994.tb06341.x. 8112310 PMC394898

[38] Botteaux A, Sory MP, Biskri L, Parsot C, Allaoui A. 2009. MxiC is secreted by and controls the substrate specificity of the *Shigella flexneri* type III secretion apparatus. Mol Microbiol 71:449–460. doi:10.1111/j.1365-2958.2008.06537.x. 19017268

[39] Fields KA, Hackstadt T. 2000. Evidence for the secretion of *Chlamydia trachomatis* CopN by a type III secretion mechanism. Mol Microbiol 38:1048–1060. doi:10.1046/j.1365-2958.2000.02212.x. 11123678

[40] Huang J, Lesser CF, Lory S. 2008. The essential role of the CopN protein in *Chlamydia pneumoniae* intracellular growth. Nature 456:112–115. doi:10.1038/nature07355. 18830244 PMC2673727

[41] Archuleta TL, Du Y, English CA, Lory S, Lesser C, Ohi MD, Ohi R, Spiller BW. 2011. The *Chlamydia* effector chlamydial outer protein N (CopN) sequesters tubulin and prevents microtubule assembly. J Biol Chem 286:33992–33998. doi:10.1074/jbc.M111.258426. 21841198 PMC3190796

[42] Nawrotek A, Guimaraes BG, Velours C, Subtil A, Knossow M, Gigant B. 2014. Biochemical and structural insights into microtubule perturbation by CopN from *Chlamydia pneumoniae*. J Biol Chem 289:25199–25210. doi:10.1074/jbc.M114.568436. 25056950 PMC4155683

[43] Holm L. 2020. Using dali for protein structure comparison. Methods Mol Biol 2112:29–42. doi:10.1007/978-1-0716-0270-6_3. 32006276

[44] Deane JE, Roversi P, King C, Johnson S, Lea SM. 2008. Structures of the *Shigella flexneri* type 3 secretion system protein MxiC reveal conformational variability amongst homologues. J Mol Biol 377:985–992. doi:10.1016/j.jmb.2008.01.072. 18304577 PMC2724173

[45] Burkinshaw BJ, Souza SA, Strynadka NC. 2015. Structural analysis of SepL, an enteropathogenic *Escherichia coli* type III secretion-system gatekeeper protein. Acta Crystallogr F Struct Biol Commun 71:1300–1308. doi:10.1107/S2053230X15016064. 26457522 PMC4601595

[46] Schubot FD, Jackson MW, Penrose KJ, Cherry S, Tropea JE, Plano GV, Waugh DS. 2005. Three-dimensional structure of a macromolecular assembly that regulates type III secretion in *Yersinia pestis*. J Mol Biol 346:1147–1161. doi:10.1016/j.jmb.2004.12.036. 15701523

[47] Holm L, Kaariainen S, Rosenstrom P, Schenkel A. 2008. Searching protein structure databases with DaliLite v.3. Bioinformatics 24:2780–2781. doi:10.1093/bioinformatics/btn507. 18818215 PMC2639270

[48] Westerhausen S, Nowak M, Torres-Vargas CE, Bilitewski U, Bohn E, Grin I, Wagner S. 2020. A NanoLuc luciferase-based assay enabling the real-time analysis of protein secretion and injection by bacterial type III secretion systems. Mol Microbiol 113:1240–1254. doi:10.1111/mmi.14490. 32068313

[49] Braet J, Catteeuw D, Van Damme P. 2022. Recent advancements in tracking bacterial effector protein translocation. Microorganisms 10. doi:10.3390/microorganisms10020260. PMC8876096 35208715

[50] Schwinn MK, Machleidt T, Zimmerman K, Eggers CT, Dixon AS, Hurst R, Hall MP, Encell LP, Binkowski BF, Wood KV. 2018. CRISPR-mediated tagging of endogenous proteins with a luminescent peptide. ACS Chem Biol 13:467–474. doi:10.1021/acschembio.7b00549. 28892606

[51] Galan JE, Lara-Tejero M, Marlovits TC, Wagner S. 2014. Bacterial type III secretion systems: specialized nanomachines for protein delivery into target cells. Annu Rev Microbiol 68:415–438. doi:10.1146/annurev-micro-092412-155725. 25002086 PMC4388319

[52] Notti RQ, Stebbins CE. 2016. The structure and function of type III secretion systems. Microbiol Spectr 4. doi:10.1128/microbiolspec.VMBF-0004-2015. PMC4804468 26999392

[53] Iriarte M, Sory MP, Boland A, Boyd AP, Mills SD, Lambermont I, Cornelis GR. 1998. TyeA, a protein involved in control of Yop release and in translocation of *Yersinia* Yop effectors. EMBO J 17:1907–1918. doi:10.1093/emboj/17.7.1907. 9524114 PMC1170537

[54] Ferracci F, Schubot FD, Waugh DS, Plano GV. 2005. Selection and characterization of *Yersinia pestis* YopN mutants that constitutively block Yop secretion. Mol Microbiol 57:970–987. doi:10.1111/j.1365-2958.2005.04738.x. 16091038

[55] Kubori T, Galan JE. 2002. *Salmonella* type III secretion-associated protein InvE controls translocation of effector proteins into host cells. J Bacteriol 184:4699–4708. doi:10.1128/JB.184.17.4699-4708.2002. 12169593 PMC135284

[56] Deane JE, Abrusci P, Johnson S, Lea SM. 2010. Timing is everything: the regulation of type III secretion. Cell Mol Life Sci 67:1065–1075. doi:10.1007/s00018-009-0230-0. 20043184 PMC2835726

[57] Younis R, Bingle LE, Rollauer S, Munera D, Busby SJ, Johnson S, Deane JE, Lea SM, Frankel G, Pallen MJ. 2010. SepL resembles an aberrant effector in binding to a class 1 type III secretion chaperone and carrying an N-terminal secretion signal. J Bacteriol 192:6093–6098. doi:10.1128/JB.00760-10. 20833800 PMC2976445

[58] Akeda Y, Galan JE. 2005. Chaperone release and unfolding of substrates in type III secretion. Nature 437:911–915. doi:10.1038/nature03992. 16208377

[59] Milne-Davies B, Helbig C, Wimmi S, Cheng DWC, Paczia N, Diepold A. 2019. Life after secretion-*Yersinia enterocolitica* rapidly toggles effector secretion and can resume cell division in response to changing external conditions. Front Microbiol 10:2128. doi:10.3389/fmicb.2019.02128. 31572334 PMC6753693

[60] Wimmi S, Balinovic A, Jeckel H, Selinger L, Lampaki D, Eisemann E, Meuskens I, Linke D, Drescher K, Endesfelder U, Diepold A. 2021. Dynamic relocalization of cytosolic type III secretion system components prevents premature protein secretion at low external pH. Nat Commun 12:1625. doi:10.1038/s41467-021-21863-4. 33712575 PMC7954860

[61] Stainer DW, Scholte MJ. 1970. A simple chemically defined medium for the production of phase I *Bordetella pertussis*. J Gen Microbiol 63:211–220. doi:10.1099/00221287-63-2-211. 4324651

[62] Hanawa T, Kamachi K, Yonezawa H, Fukutomi T, Kawakami H, Kamiya S. 2016. Glutamate limitation, BvgAS activation, and (p)ppGpp regulate the expression of the *Bordetella pertussis* type 3 secretion system. J Bacteriol 198:343–351. doi:10.1128/JB.00596-15. 26527639 PMC4751785

[63] Kurushima J, Kuwae A, Abe A. 2012. Iron starvation regulates the type III secretion system in *Bordetella bronchiseptica*. Microbiol Immunol 56:356–362. doi:10.1111/j.1348-0421.2012.00442.x. 22376189

[64] Gibson DG, Young L, Chuang RY, Venter JC, Hutchison CA, 3rd, Smith HO. 2009. Enzymatic assembly of DNA molecules up to several hundred kilobases. Nat Methods 6:343–345. doi:10.1038/nmeth.1318. 19363495

[65] Kabsch W. 2010. Xds. Acta Crystallogr D Biol Crystallogr 66:125–132. doi:10.1107/S0907444909047337. 20124692 PMC2815665

[66] Holubova J, Kamanova J, Jelinek J, Tomala J, Masin J, Kosova M, Stanek O, Bumba L, Michalek J, Kovar M, Sebo P. 2012. Delivery of large heterologous polypeptides across the cytoplasmic membrane of antigen-presenting cells by the *Bordetella* RTX hemolysin moiety lacking the adenylyl cyclase domain. Infect Immun 80:1181–1192. doi:10.1128/IAI.05711-11. 22215742 PMC3294662

[67] McCoy AJ, Grosse-Kunstleve RW, Adams PD, Winn MD, Storoni LC, Read RJ. 2007. Phaser crystallographic software. J Appl Crystallogr 40:658–674. doi:10.1107/S0021889807021206. 19461840 PMC2483472

[68] Thorn A, Sheldrick GM. 2013. Extending molecular-replacement solutions with SHELXE. Acta Crystallogr D Biol Crystallogr 69:2251–2256. doi:10.1107/S0907444913027534. 24189237 PMC3817699

[69] Sammito M, Millan C, Rodriguez DD, de Ilarduya IM, Meindl K, De Marino I, Petrillo G, Buey RM, de Pereda JM, Zeth K, Sheldrick GM, Uson I. 2013. Exploiting tertiary structure through local folds for crystallographic phasing. Nat Methods 10:1099–1101. doi:10.1038/nmeth.2644. 24037245

[70] Cowtan K. 2006. The Buccaneer software for automated model building. 1. Tracing protein chains. Acta Crystallogr D Biol Crystallogr 62:1002–1011. doi:10.1107/S0907444906022116. 16929101

[71] Murshudov GN, Skubak P, Lebedev AA, Pannu NS, Steiner RA, Nicholls RA, Winn MD, Long F, Vagin AA. 2011. REFMAC5 for the refinement of macromolecular crystal structures. Acta Crystallogr D Biol Crystallogr 67:355–367. doi:10.1107/S0907444911001314. 21460454 PMC3069751

[72] Emsley P, Lohkamp B, Scott WG, Cowtan K. 2010. Features and development of Coot. Acta Crystallogr D Biol Crystallogr 66:486–501. doi:10.1107/S0907444910007493. 20383002 PMC2852313

[73] Williams CJ, Headd JJ, Moriarty NW, Prisant MG, Videau LL, Deis LN, Verma V, Keedy DA, Hintze BJ, Chen VB, Jain S, Lewis SM, Arendall WB, 3rd, Snoeyink J, Adams PD, Lovell SC, Richardson JS, Richardson DC. 2018. MolProbity: more and better reference data for improved all-atom structure validation. Protein Sci 27:293–315. doi:10.1002/pro.3330. 29067766 PMC5734394

[74] Krissinel E, Henrick K. 2007. Inference of macromolecular assemblies from crystalline state. J Mol Biol 372:774–797. doi:10.1016/j.jmb.2007.05.022. 17681537

[75] Schindelin J, Arganda-Carreras I, Frise E, Kaynig V, Longair M, Pietzsch T, Preibisch S, Rueden C, Saalfeld S, Schmid B, Tinevez JY, White DJ, Hartenstein V, Eliceiri K, Tomancak P, Cardona A. 2012. Fiji: an open-source platform for biological-image analysis. Nat Methods 9:676–682. doi:10.1038/nmeth.2019. 22743772 PMC3855844

