# Supplementary material for: BopN is a Gatekeeper of the Bordetella Type III Secretion System
Source: Microbiol Spectr. 2023 Apr 10;11(3):e04112-22. doi: 10.1128/spectrum.04112-22 (PMC10269732; doi:10.1128/spectrum.04112-22)
Supplement: Supplemental file 1 — Supplemental material. Download spectrum.04112-22-s0001.pdf, PDF file, 2.9 MB [file spectrum.04112-22-s0001.pdf]

Supporting figures:

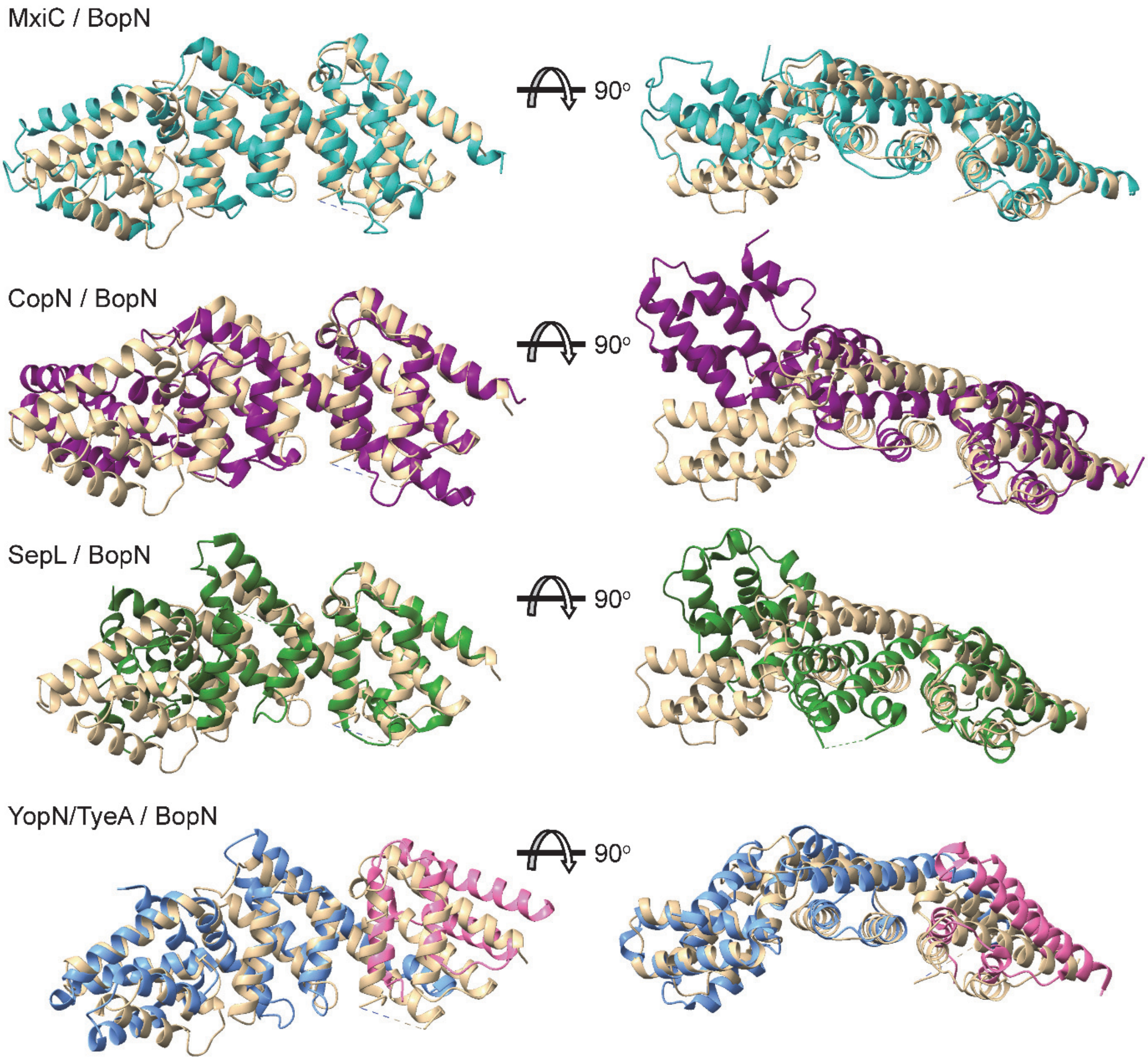

Figure S1. Superposition of the BopN protein on structurally characterized members of the SctW family. BopN is colored brown and was superposed on *Shigella* MxiC (light see green, Protein Data Bank code 2VJ5-B), *Chlamydia* CopN (purple, Protein Data Bank code 6GX7-H), *E. coli* SepL (forest green, Protein Data Bank code 5C9E-B) and *Yersinia* YopN (cornflower blue**,** Protein Data Bank code 1XL3-B) and TyeA (hot pink, Protein Data Bank code 1XL3-D). This figure was generated by Chimera X1. 5.

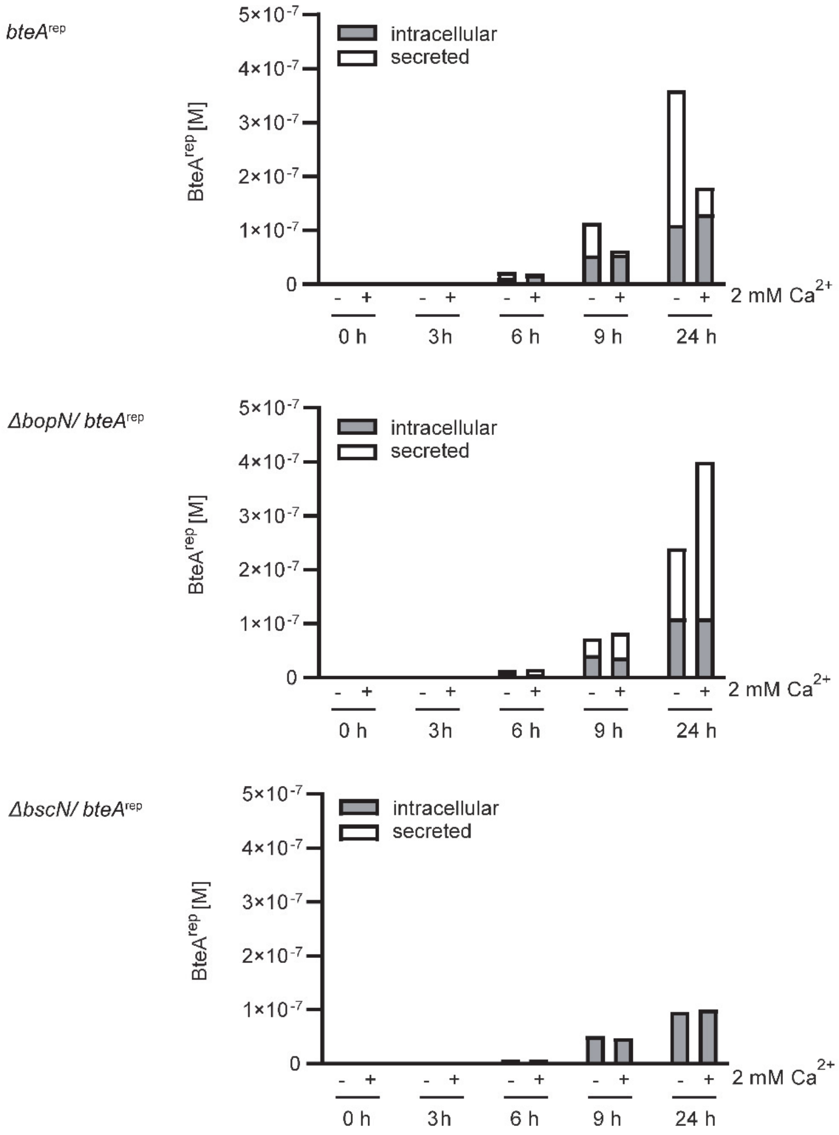

Figure S2. BopN controls secretion of BteA. The amounts of intracellular and secreted BteA^{rep} in cultures grown in the presence or absence of 2 mM Ca^{2+} were determined from the luminescence signal. The recombinant BteA^{rep} protein was used for calibration. Data from a representative experiment out of 2 are shown.

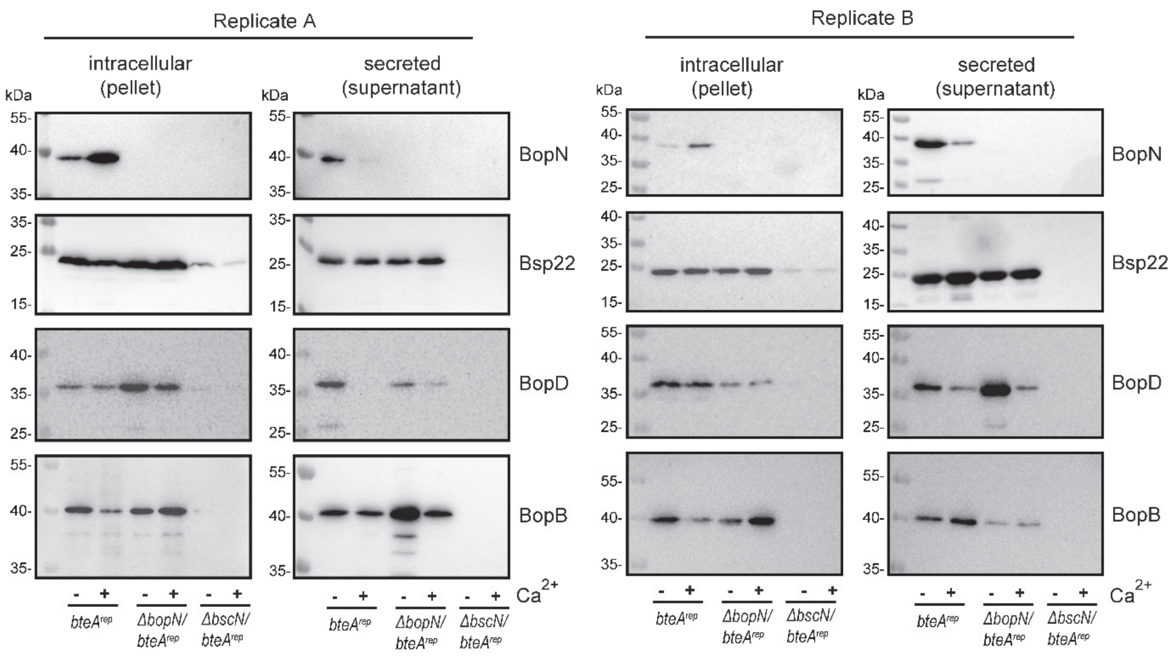

Figure S3. BopN does not regulate secretion of the tip protein Bsp22 and translocators BopD and BopB. Supernatants and pellets of overnight cultures of the *bteA*^{rep} strain and its derivatives were analyzed by immunoblotting with anti-BopN (BopN, 39 kDa), anti-Bsp22 (Bsp22, 22 kDa), anti-BopD (BopD, 32 kDa), and anti-BopB (BopB, 40 kDa) antisera. Two replicate experiments show that BopN protein does not prevent secretion of BopD and BopB translocators. However, while the trends of BopN and Bsp22 detection in cell culture fractions (intracellular *versus* secreted) were highly consistent and reproducible, a large variation in amounts of detected BopB and BopD between fractions was observed. The BopD protein, 32 kDa, migrates higher than expected, which has been observed previously (1).

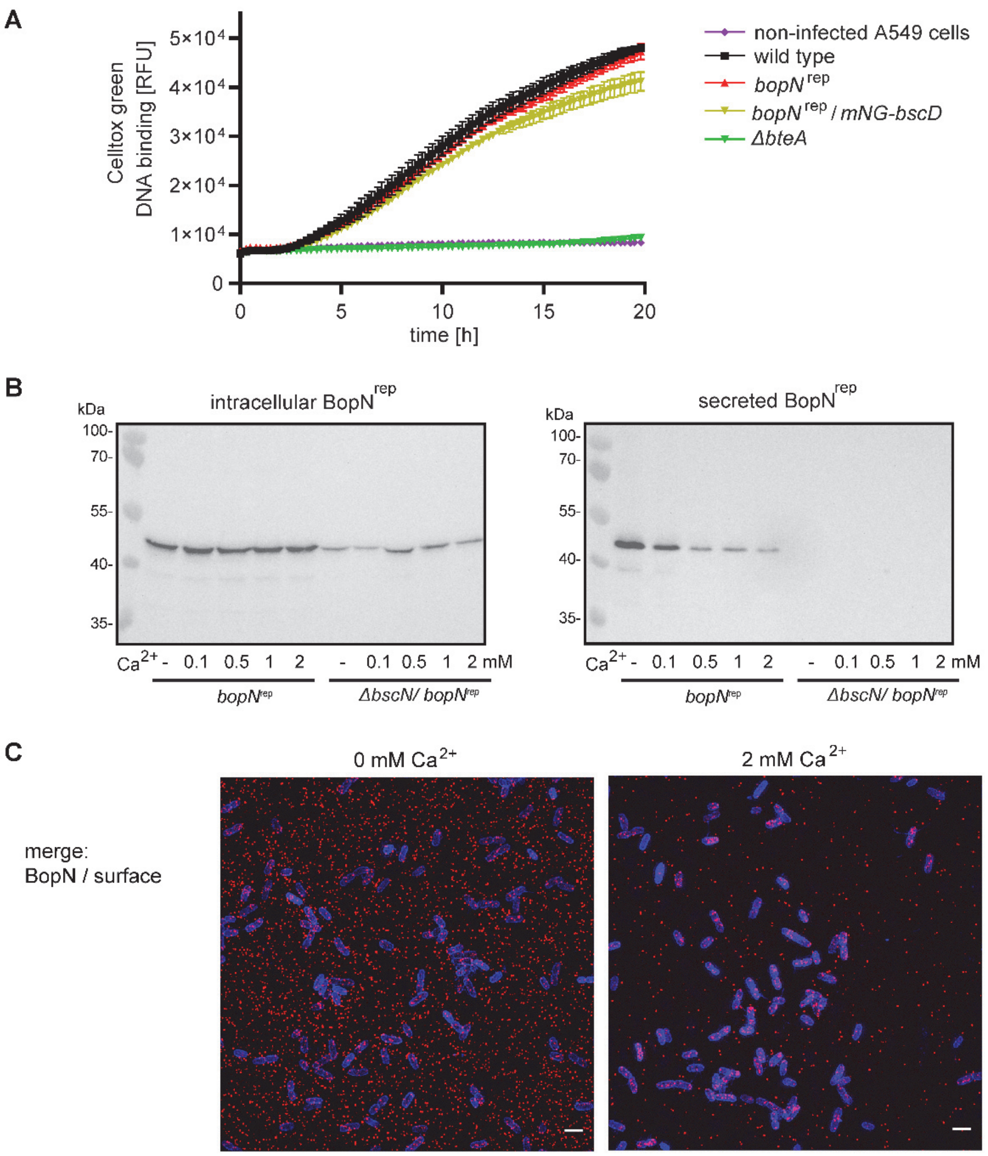

Figure S4. BopN responds to low Ca^{2+} concentration.

(A) Tagging of the BopN or BscD proteins does not affect the functionality of the *Bordetella* injectisome. Human lung epithelial A549 cells were infected with *B. bronchiseptica* wild type and derivative strains at MOI 5:1. Cytotoxicity was measured as real-time kinetics of membrane permeabilization and determined with the fluorescent DNA binding dye CellTox Green. Shown are mean values ± SD of triplicate wells from a representative experiment. Data are representative of two independent experiments.

(B-C) Calcium-rich medium prevents BopN secretion. In **(B)** cells of the *bopN*^{rep} and the secretion-deficient Δ*bscN*/ *bopN*^{rep} derivative were incubated at the indicated concentration of Ca^{2+} for 90 min. The amounts of intracellular and secreted BopN^{rep}, 45 kDa, were determined by immunoblot detection. Molecular weight marker is shown. In **(C)** cells of b*opN*^{rep} were incubated for 90 min in the absence or presence of 2 mM Ca^{2+}. After fixation, BopN^{rep} was visualized with an anti-SPOT nanobody conjugated to ATTO594, while the outer surface of *Bordetella* was stained with a rabbit anti-*Bordetella* serum followed by anti-rabbit IgG-DyLight 405 conjugate. Z-max projections are shown. The fluorescence images shown are representative of 3 independent experiments. Bars represent 2 µm.

Supporting tables:

Table S1. List of bacterial strains used in this study.

Strain	Genotype and relevant description	Reference
E. coli		
XL1-Blue	*rec*A1 *end*A1 *gyr*A96 *thi*-1 *hsd*R17 *sup*E44 *rel*A1 *lac* F´ *pro*AB *lacIq*ZΔM15 Tn10 Tet^r	Stratagene
SM10 λpir	*thi thr leu ton*A *lac*Y *sup*E *rec*A::RP4-2-Tc::Mu Km λpir	(2, 3)
BL21 λ(DE3)	F– *omp*T $hsdS_B$ (rB–, mB–) *gal dcm* (DE3)	Novagen
B. bronchiseptica		
WT	*Bb*RB50 WT; wild type *Bordetella bronchiseptica* RB50 (B1976); complex I rabbit isolate; ST-12	(4, 5)
mNG-bscD	BbRB50 WT derivative with *bscD* allele fused to mNeonGreen (mNG) with GSSGGSSG linker at N-terminus	This study
Δ*bopN*	*BbRB50* WT derivative with *bopN* deletion of codons M1-A364, start codon of BB1615 (orf4/sycN) was maintained	This study
Δ*bteA*	*Bb*RB50 WT derivative with *bte*A in-frame deletion of codons L2-A657	This study
Δ*bopN*/ Δ*bteA*	Δ*bteA* derivative with *bopN* deletion of codons M1-A364, start codon of BB1615 (orf4/sycN) was maintained	This study
bteA^{rep}	*BbRB50* WT encoding *bteA* codons M1-V130 fused to HiBiT with GSSG linker at C-terminus, *bteA* codons K131-A657 were removed, STOP codon is retained	This study
ΔbopN/ bteA^{rep}	*bteA*^{rep} derivative with *bopN* deletion of codons M1-A364, start codon of BB1615 *(orf4/sycN)* was maintained	This study
ΔbscN/ bteA^{rep}	*bteA*^{rep} derivative with *bscN* in-frame deletion of codons R2-E443	This study
Δbsp22/ bteA^{rep}	*bteA*^{rep} derivative with *bsp22* in-frame deletion of codons T10-S198	This study
bopN^{rep}	*Bb*RB50 WT derivative with *bopN* allele fused to HiBiT-3xFLAG-SPOT with GSSG linker at C-terminus	This study
ΔbscN/ bopN^{rep}	*bopN*^{rep} derivative with *bscN* in-frame deletion of codons R2-E443	This study
mNG-bscD/bopN^{rep}	*bopN*^{rep} derivative with *bscD* allele fused to mNeonGreen (mNG) with GSSGGSSG linker at N-terminus	This study
bopN^{rep}/ Δ*bteA*	*bopN*^{rep} derivative with *bteA* in-frame deletion of codons L2-A657	This study
Δbsp22/ bopN^{rep}/ Δ*bteA*	*bopN*^{rep}/ Δ*bteA* derivative with *bsp22* in-frame deletion of codons T10-S198	This study
ΔbscN/ bopN^{rep}/ Δ*bteA*	*bopN*^{rep}/ Δ*bteA* derivative with *bscN* in-frame deletion of codons R2-E443	This study

Table S2. List of plasmids used in this study.

Plasmid	Description	Reference
pET28b	6xHis-tagging expression vector *E. coli,* T7 promoter, KmR	Novagen
pET28b *Bp*TohamaI BopN	pET28b vector encoding BopN protein (aa 1-365) of *B. pertussis* TohamaI fused to 6xHis tag on its N-terminus	This study
pGEX-6P1	GST-tagging expression vector for *E. coli,* lac operator, AmpR	GE Healthcare
pGEX-6P1 *Bb*RB50 $BopN^{rep}$	pGEX-6P1 vector encoding BopN protein of *Bb*RB50 fused to HiBiT-3xFLAG-SPOT with GSSG linker at C-terminus	This study
pGEX-6P1 *Bb*RB50 $BteA^{rep}$	pGEX-6P1 vector encoding aa 1-130 of BteA effector of *Bb*RB50 fused to HiBiT with GSSG linker at C-terminus	This study
pSS4245	Allelic exchange vector for *Bordetella spp.*, contains *ptx* promoter, *I-SceI*, *oriV*, *AmpR, StrR, KmR, BleR, TetR* and an *I-SceI* cleavage site for counterselection	(6, 7)
pSS4245 *Bb*RB50 *mNG-bscD*	pSS4245 vector containing homology regions h1 (627 bp, 1717770 -1718396) and h2 (556 bp, 1717214-1717769) flanking the N-terminus of *bscD* of *BbRB50,* with intervening insertions of codons coding for mNeonGreen and GSSGGSSG linker	This study
pSS4245 *Bb*RB50 Δ*bscN*	pSS4245 vector containing homology regions h1 (681bp, 1728767 -1729447) and h2 (658 bp, 1730777-1731434) flanking in-frame deletion of codons R2-E443 in the *bscN* of *Bb*RB50	This study
pSS4245 *Bb*RB50 Δ*bteA*	pSS4245 vector containing homology regions h1 (712 bp, 4501345 -4502056) and h2 (622 bp, 4504028-4504649) flanking in-frame deletion of codons L2-A657 in the *bteA* of *Bb*RB50	This study
pSS4245 *Bb*RB50 Δ*bopN*	pSS4245 vector containing homology regions h1 (713 bp, 1722120 -1722832) and h2 (749 bp, 1720277-1721025) flanking deletion of codons M1-A364 in the *bopN* of *Bb*RB50	This study
pSS4245 *Bb*RB50 Δ*bsp22*	pSS4245 vector containing homology regions h1 (769 bp, 1721541 -1722309) and h2 (740 bp, 1722877-1723616) flanking in-frame deletion of codons T10-S198 in the *bsp22* of *Bb*RB50	This study
pSS4245 *Bb*RB50 $bopN^{rep}$	pSS4245 vector containing homology regions h1 (750 bp, 1721028 -1721777) and h2 (756 bp, 1720272-1721027) flanking the C-terminus of the *bopN* allele of *Bb*RB50, with intervening insertion of codons coding for HiBiT-3xFLAG-SPOT and GSSG linker	This study
pSS4245 *Bb*RB50 $bteA^{rep}$	pSS4245 vector containing homology regions h1 (654 bp, 4501790 -4502443) and h2 (720 bp, 4504028-4504747) of the *bteA* allele of *Bb*RB50, with intervening insertion of codons coding for HiBiT and GSSG linker	This study
pLJM1-FLAG-LgBit	pLJM vector encoding LgBit fused to FLAG with GSSGGGGSGGGGSSG linker on its N-terminus	This study
pCMV-VSV-G	Vector encoding envelope protein for producing lentiviral and MuLV retroviral particles, Addgene item #8454	(8)
psPAX2	2nd generation lentiviral packaging plasmid, Addgene item #12260	Addgene

References

1. Nogawa H, Kuwae A, Matsuzawa T, Abe A. 2004. The type III secreted protein BopD in Bordetella bronchiseptica is complexed with BopB for pore formation on the host plasma membrane. J Bacteriol 186:3806-13.
2. Simon R, Priefer U, Pühler A. 1983. A Broad Host Range Mobilization System for In Vivo Genetic Engineering: Transposon Mutagenesis in Gram Negative Bacteria. Bio/Technology 1:784.
3. Skopova K, Tomalova B, Kanchev I, Rossmann P, Svedova M, Adkins I, Bibova I, Tomala J, Masin J, Guiso N, Osicka R, Sedlacek R, Kovar M, Sebo P. 2017. Cyclic AMP-Elevating Capacity of Adenylate Cyclase Toxin-Hemolysin Is Sufficient for Lung Infection but Not for Full Virulence of Bordetella pertussis. Infect Immun 85.
4. Cotter PA, Miller JF. 1994. BvgAS-mediated signal transduction: analysis of phase-locked regulatory mutants of Bordetella bronchiseptica in a rabbit model. Infect Immun 62:3381-90.
5. Diavatopoulos DA, Cummings CA, Schouls LM, Brinig MM, Relman DA, Mooi FR. 2005. Bordetella pertussis, the causative agent of whooping cough, evolved from a distinct, human-associated lineage of B. bronchiseptica. PLoS Pathog 1:e45.
6. Inatsuka CS, Xu Q, Vujkovic-Cvijin I, Wong S, Stibitz S, Miller JF, Cotter PA. 2010. Pertactin is required for Bordetella species to resist neutrophil-mediated clearance. Infect Immun 78:2901-9.
7. Posfai G, Kolisnychenko V, Bereczki Z, Blattner FR. 1999. Markerless gene replacement in Escherichia coli stimulated by a double-strand break in the chromosome. Nucleic Acids Res 27:4409-15.
8. Stewart SA, Dykxhoorn DM, Palliser D, Mizuno H, Yu EY, An DS, Sabatini DM, Chen IS, Hahn WC, Sharp PA, Weinberg RA, Novina CD. 2003. Lentivirus-delivered stable gene silencing by RNAi in primary cells. RNA 9:493-501.
